# *In vitro* vascularization of 3D cell aggregates in microwells with integrated vascular beds

**DOI:** 10.1016/j.mtbio.2024.101260

**Published:** 2024-09-19

**Authors:** Maria G. Fois, Zeinab N. Tahmasebi Birgani, Carmen López-Iglesias, Kèvin Knoops, Clemens van Blitterswijk, Stefan Giselbrecht, Pamela Habibović, Roman K. Truckenmüller

**Affiliations:** a Department of Instructive Biomaterials Engineering, MERLN Institute for Technology-Inspired Regenerative Medicine, Faculty of Health, Medicine and Life Sciences, Maastricht University, Universiteitssingel 40, 6229 ER, Maastricht, the Netherlands; b Microscopy CORE Lab, Faculty of Health, Medicine and Life Sciences, Maastricht University, Universiteitssingel 50, 6229 ER, Maastricht, the Netherlands

**Keywords:** 3D cell culture, Spheroids, Vascularization, Endothelial cells, Microwells, Microthermoforming

## Abstract

Most human tissues possess vascular networks supplying oxygen and nutrients. Engineering of functional tissue and organ models or equivalents often require the integration of artificial vascular networks. Several approaches, such as organs on chips and three-dimensional (3D) bioprinting, have been pursued to obtain vasculature and vascularized tissues *in vitro*. This technical feasibility study proposes a new approach for the *in vitro* vascularization of 3D microtissues. For this, we thermoform arrays of round-bottom microwells into thin non-porous and porous polymer films/membranes and culture vascular beds on them from which endothelial sprouting occurs in a Matrigel-based 3D extra cellular matrix. We present two possible culture configurations for the microwell-integrated vascular beds. In the first configuration, human umbilical vein endothelial cells (HUVECs) grow on and sprout from the inner wall of the non-porous microwells. In the second one, HUVECs grow on the outer surface of the porous microwells and sprout through the pores toward the inside. These approaches are extended to lymphatic endothelial cells. As a proof of concept, we demonstrate the *in vitro* vascularization of spheroids from human mesenchymal stem cells and MG-63 human osteosarcoma cells. Our results show the potential of this approach to provide the spheroids with an abundant outer vascular network and the indication of an inner vasculature.

## Graphical abstract

**Figure:**
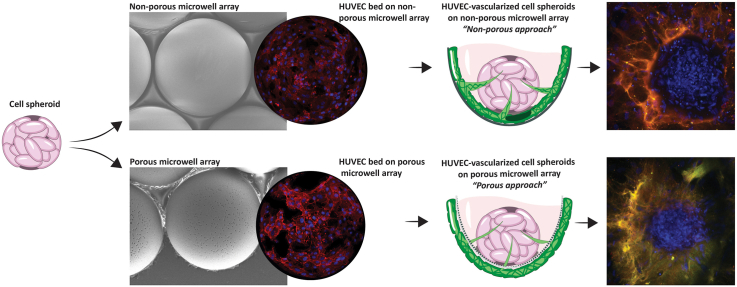

## 1 Introduction

With some exceptions including articular cartilage [1] and the cornea [2], the vast majority of native tissues and organs rely on the presence of an intricate vascular network to sustain oxygen and nutrient supply [3]. In vascularized tissues, the diffusion limit of oxygen requires that the cells are located within 100–200 μm of blood vessels [4]. In tissue engineering (TE), the development of vascularization strategies is imperative to obtain functional tissue or organ equivalents. From a therapeutic perspective, there has been great progress in the development of functional grafts by adding angiogenic factors prior to implantation [5]. To promote endothelialization and a good interaction with the host, engineered vascular grafts have been functionalized with immobilized angiogenic factors, such as the vascular endothelial growth factor (VEGF) [6] and other endothelial-affine peptides [7]. On the other hand, considerations regarding the physical characteristics of the graft, such as its porosity, have also considerably improved the interaction with infiltrating cells [8]. Efforts toward the development of functional vasculature *in vitro* have included several tissue engineering approaches, such as corresponding organ-on-chip (OoC) technologies [9,10], or of additive manufacturing technologies, for example three-dimensional (3D) bioprinting [[11], [12], [13]].

Cell aggregates, such as spheroids, are self-organizing 3D microtissues exhibiting realistic cell-cell and cell-matrix contacts as well as a close-to-native spatial-temporal signaling [14]. In the past decades, spheroids served as injectable cell-based therapies [15,16], have been extensively used to model cancer microenvironments *in vitro* [[17], [18], [19]] and have been often chosen to model 3D cell behavior and interactions in fundamental regeneration studies [[20], [21], [22]], to name a few applications. Although cell spheroids are a very popular option in TE, they undoubtedly have shortcomings when it comes to the generation of big(ger) tissue units, such as their typical multi-layered structure, which is characterized by a central necrotic core, depleted of oxygen, nutrients and growth factors [23]. This phenomenon occurs in a spheroid size-dependent manner and ultimately compromises cell survival and behavior. A possible solution to this is the integration of a functional and therefore also perfusable vascular network to nourish the spheroids, particularly the large ones. Efforts in this direction approached the vascularization of spheroids through direct co-cultures of (cells basically forming the) spheroids with endothelial cells in different ratios [24,25]. Compartmentalization-based directed co-culture strategies have also proven to promote a good interaction between endothelial cells and spheroids in terms of viability and functionality. As an example, a reported approach consisted in the vascularization of spheroids on chip through an artificial vascular network based on microfluidic channels [26]. Alternatively, the use of hydrogel-embedded spheroids in conjunction with 3D bioprinting allowed the formation of spatially organized vascularized spheroids [27]. Despite the enormous progress in this field, there is still the need of methods and, consequently, culture platforms to vascularize 3D cell aggregates, such as spheroids, with perfusable vascular networks [28]. 3D *in vitro* models, which are able to recapitulate vascularization mechanisms in a time-effective and reliable manner, can provide a useful compromise between biomimetic cell milieu and external control.

In this context, spheroid formation and culture in microwells have emerged as a frequently adopted method, featuring several types of used microwells, such as (micro)thermoformed [29], agarose-casted [30], photo-patterned [31] and laser-ablated ones [32]. Most of the strategies that apply microwells for spheroid vascularization rely on the physical confinement exerted by the microwell geometry on the cells, which consequently self-assemble into multicellular, vascularized 3D aggregates supported by gravity and proximity [[33], [34], [35]]. On the other hand, to the best of our knowledge, there has been no study yet that reports microwell walls as cell culture substrates in a spheroid vascularization approach. The use of the walls of microwells as adhesive surfaces for endothelial cells to form vascular beds surrounding spheroids seeded into the microwells represents a novel concept of a microwell-based co-culture for the *in vitro* vascularization of spheroids. Advantages of this approach may include a more straightforward access to early endothelial cell-spheroid interactions, as the respective cell population is initially separated. This is in contrast to multicellular spheroids where from the very beginning the two cell populations are confined within the same microconstruct. Moreover, the chemical and structural properties of the microwells can be tailored to influence cell behavior and interactions [[36], [37], [38], [39], [40]]. This includes the introduction of porosity in the microwells' walls using our 3D ion track etch technology.

**Fig. 1:**
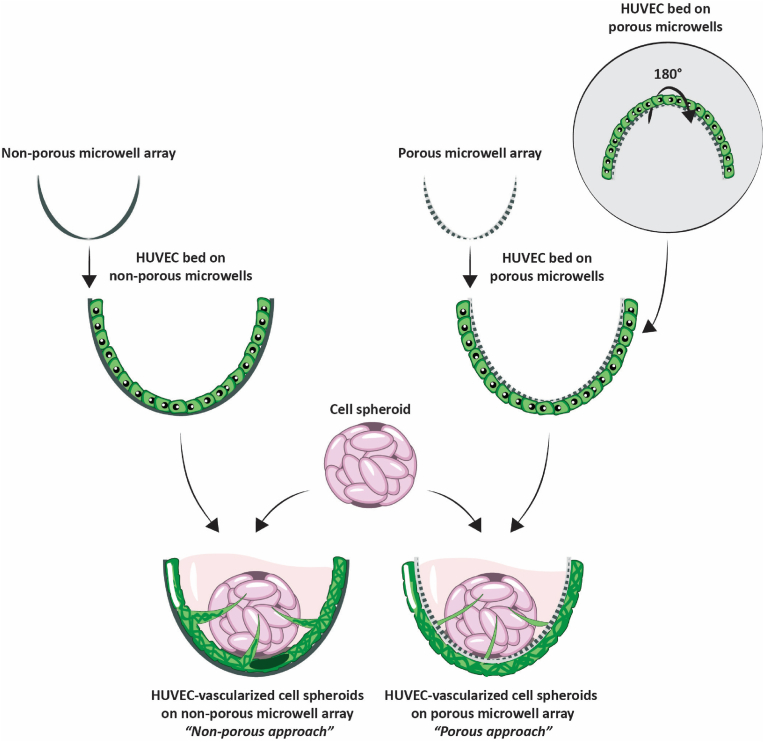
**HUVEC-based spheroid vascularization on microwell arrays:** Schematic representation of the proposed spheroid vascularization *in vitro* model. The vascularization was achieved through the culture of HUVECs (displayed in green) on the surface of microwells in two different configurations. In the first one, HUVECs were seeded onto the inner surface of non-porous microwells and allowed to form a monolayer (top left). In the second configuration, HUVECs were seeded onto the outer surface of porous microwells (top right). For this, the microwell arrays were temporarily flipped upside down. hMSC spheroids (displayed in pink) were pre-formed by physical confinement in non-adherent microwells prior to their co-culture with HUVECs. For non-porous microwells, the spheroid and the HUVEC lining co-exist within the same space. For porous microwells, they are separated by the microwell walls and sprouting of the vascular network and its contact with the spheroid occurs through the pores of the microwell.

The aim of this technical study was to develop a novel *in vitro* vascularization approach based on the culture of endothelial cells, here exemplarily human umbilical vein endothelial cells (HUVECs), on arrays of thin-walled microwells fabricated from non-porous and porous polymer films (Fig. 1). The fabrication was by microscale thermoforming [36], the material were thin, highly transparent polycarbonate (PC) films/membranes, allowing for high-content and high-resolution imaging. The HUVECs were seeded onto the inner or outer surface of the non-porous or porous microwells, respectively. Thereby, the HUVECs formed a vascular bed, from which the sprouting of a vascular network occurred, directly or through the pores, into the inner space of the microwells. The non-porous variant of the microwells is cheaper to fabricate and does not require a seeding of the HUVECs from the microwells' backside. On the other hand, the porous microwell version allows more complex culture configurations. Its microwell walls can serve as tunable permeable physical barrier between the spheroid and the vascular bed by varying pore diameter and density. In track etch technology, these two parameters can be adjusted independently. According to the selected porosity, the porous microwells can enable an initially either more direct or indirect co-culture between the endothelial cells outside the microwell and a target spheroid inside the microwell. Furthermore, the porous microwells can allow the formation of gradients of soluble factors across their walls. Finally, if the vascular network would be perfusable, there could be microflows from outside the microwells through their inside, there within the lumen of the microvessels, and back to the microwells' outside. We first demonstrated the feasibility of the self-assembly of a 3D vascular network on non-porous and porous microwell arrays in culture medium-diluted Matrigel. In the case of the porous microwells, the optimal pore diameter and density to support both the formation of a curved vascular bed on the outer microwell surface and, subsequently, their 3D sprouting through the pores was determined. Secondly, as a proof of concept, we co-cultured the vascular beds integrated into the microwells with human mesenchymal stem/stromal cell (hMSC) spheroids seeded into the microwells together with diluted Matrigel. We then assessed the spheroids' vascularization within the microwells in terms of expression of a selection of vascular endothelial markers and characterizing the extension of the sprouting network. To showcase the potential of the proposed approach, we further evaluated the feasibility of promoting lymphangiogenesis of human dermal lymphatic endothelial cells (hDLECs) on the microwell arrays, as well as we investigated the vascularization of spheroids from MG-63 human osteosarcoma cells.

## 2 Materials and methods

### 2.1 Fabrication, characterization and cell culture preparation of the microwell arrays

Arrays of circular round-/U-bottom microwells were fabricated by free-forming variant of gas-assisted microthermoforming [36,37]. Hereby, two different micromilled brass molds were used containing nine hexagonal and twelve rectangular arrays of 289 and 30 hexagonally arranged circular-cylindrical microcavities, respectively, in both cases with a diameter of 550 μm.

For the fabrication of the non-porous microwell arrays, a dense 50 μm thick PC film (it4ip) was formed as previously described by us [29], which was at a temperature of 154 °C and pressure of 20 bar. After 30 s of applying the forming pressure, the heating was stopped and the film was allowed to cool down to 100 °C. At this point, the pressure was released and the formed film demolded.

For the porous microwell arrays, four ion track-etched PC films (it4ip) were selected, which presented a nominal pore diameter of 0.2, 0.4, 0.8 and 2 μm. The porous films had a thickness of 48–49 μm and a pore density of 10^6^ pores cm^−2^. To form the porous microwells, a procedure similar to the one previously described by us [41] was applied. Thereby, the above-listed porous PC films were formed together with a non-porous 50 μm thick polypropylene (PP) film (Durable) before the forming laminated on the PC film to seal them against the pressure gas leaking through their pores. A pre pressure of 1.5 bar was applied to the double-layer until the forming temperature of 154 °C was reached and a forming pressure of 20 bar was applied. The demolding was again 100 °C After this, the PP film was peeled off from the PC film and discarded.

Visual inspection of the microwell arrays was performed via scanning electron microscopy (SEM; JSM-IT200; JEOL) at various magnifications, using an accelerating voltage of 10 kV and a working distance of 10 mm. To this end, the microwells-arrays were first sputter-coated with a thin layer of gold (SC7620; Quorum Technologies). Qualitative characterization of microwells' geometrical features including the pore diameters was assessed via confocal laser scanning profilometry (VK-X200; KEYENCE).

Quantitative measurements of the microwells' (maximum) outer diameter and depth were performed in 6 microwells distributed over 2 films in each of which multiple microwell arrays (5 × 6 for the 30-microwell arrays) were formed before their separation into individual arrays. For the porous arrays, we also measured the diameters of the pores after thermoforming, which increase as a consequence of the fact that thermoforming is a stretch forming process [41]. The measurements were performed on 40 pores in 6 microwells distributed over 2 films. Here, only pores located at the bottom/apex of the microwells were measured as in this region their size and shape in the laser profilometer image used for measuring was hardly affected by the still only slightly non-perpendicular angle between the profilometer's optical axis and the curved surface of the microwells. Furthermore, the maximum stretch of the film occurs at the bottom of the microwells and it decreases toward the flat ridge between the microwells [41], making the size of the pores at the bottom a good approximation of the maximum size of the microwells' pores.

The thermoformed 289- and 30-microwell arrays were mounted at the bottom of the wells of 24- and 96-well plates, respectively, using O-rings (ERIKS). Except for pre-forming and -culturing the hMSC or MG-63 (cell) spheroids, which was done in non-porous 289-microwell arrays, in all other cases, the 30-microwell arrays were used. Non-porous microwell arrays were placed with the concave inner bottom of the microwells facing up, while the porous microwells were placed upside down, with the convex outer bottom of the microwells facing up, resembling a dome. A descending isopropanol-water dilution series, 100 %, 70 %, 50 %, 20 % and 0 % IPA in ultrapure, sterile water (Milli-Q; Millipore) was applied to wet the microwells and sterilize them as well as the O-rings prior to their use for the cell culture.

### 2.2 Cell subculture

HUVECs, isolated from the vein of the human umbilical cord (pooled from multiple donors), were purchased (PromoCell; catalog number: C-12203), expanded according to manufacturer's instruction, sub-cultured until passage 3 and kept frozen in liquid nitrogen until the experiments. At passage 4, the cells were thawed, seeded at a density of 5000 cells cm^−2^ into a T75 tissue culture flask in a ready-to-use Endothelial Cell Growth Medium 2 (EGM2; PromoCell) and cultured at 37 °C and 5 % CO_2_. The EGM2 was supplemented with 100 U/mL penicillin and 100 μg/mL streptomycin (Thermo Fisher Scientific, Gibco).

hDLECs, isolated from the dermis of human adult skin, were purchased (PromoCell; catalog number: C-12217), expanded according to manufacturer's instruction, sub-cultured until passage 5 and kept frozen in liquid nitrogen until the experiments. At passage 6, the cells were thawed, seeded at a density of 20,000 cells cm^−2^ into a T75 tissue culture flask in an ready-to-use Endothelial Cell Growth Medium MV 2 (EMV2; PromoCell) and cultured at 37 °C and 5 % CO_2_.

hMSCs, isolated from normal (non-diabetic) human adult bone marrow, were purchased (Lonza; catalog number: PT-2501; lot: 19TL329433), expanded according to manufacturer's instruction, sub-cultured until passage 2 and kept frozen in liquid nitrogen until the experiment. At passage 3, the cells were thawed, seeded at a density of 2000 cells cm^−2^ into a T225 tissue culture flask in a growth medium and cultured at 37 °C and 5 % CO_2_. The growth medium was composed of α-Minimum Essential Medium (α-MEM; Thermo Fisher Scientific, Gibco) supplemented with 10 % fetal bovine serum (FBS; Sigma-Aldrich; lot BCBX5318), 0.2 mM L-ascorbic acid 2-phosphate sesquimagnesium salt hydrate (Sigma-Aldrich), and 100 U/mL penicillin and 100 μg/mL streptomycin (Thermo Fisher Scientific, Gibco).

MG-63 human osteosarcoma cells (ATCC; CRL-1427TM) at passage 97 were thawed, seeded at a density of 5000 cells cm^−2^ into a T225 tissue culture flask in a growth medium and cultured at 37 °C and 5 % CO_2_. The growth medium was composed of α-MEM (Thermo Fisher Scientific, Gibco) supplemented with 10 % FBS (Sigma-Aldrich; lot BCBX5318), 0.2 mM 2-phosphate sesquimagnesium salt hydrate (Sigma-Aldrich), and 100 U/mL penicillin and 100 μg/mL streptomycin (Thermo Fisher Scientific, Gibco).

### 2.3 HUVEC culture on the microwell arrays and sprouting characterization

On the day of the experiment, porous and non-porous 30-microwell arrays were coated with a 1 % v/v solution of gelatin (Sigma-Aldrich) in sterile water (Milli-Q; Millipore) for 30 min. The arrays were rinsed twice with sterile Dulbecco's phosphate buffered saline (DPBS; Sigma-Aldrich) and kept in DPBS until further use. HUVECs were trypsinized and re-suspended in fresh EGM2 (PromoCell). HUVECs at passage 5 were seeded onto the inner and outer surface of the non-porous and porous microwells, respectively, at a density of 100,000 cells per microwell array and kept in culture at 37 °C and 5 % CO_2_ for 4 days. The EGM2 was refreshed at day 3. After day 4, the upside-down porous microwells were flipped back. The EGM2 was substituted with 50 % v/v Matrigel (Corning) in EGM2.

The culture of the HUVECs on the microwell arrays with diluted Matrigel and the cells' ability to promote new sprouting was evaluated after 1 and 3 days of culture by immunostaining of relevant vascular endothelial markers. To this end, the HUVECs were fixed with a 2 % v/v solution of formaldehyde (Sigma-Aldrich) in DPBS for 1 h at 37 °C. Afterwards, the cells were permeabilized with a 0.01 % v/v solution of Triton-X100 (Sigma-Aldrich) in DPBS for 30 min. A blocking step for unspecific binding was performed using a casein-based blocking serum (CAS-Block Histochemical Reagent; Thermo Fisher Scientific). The cells were incubated with a 0.1 % bovine serum albumin (BSA; VWR) containing a dilution of the primary antibodies. For this, the following antibodies were used: A sheep anti-cluster of differentiation (CD)31/platelet endothelial cell adhesion molecule (PECAM)-1 (R&D Systems), a rabbit anti-laminin (Sigma-Aldrich) and a goat anti-podocalyxin (R&D Systems), all at a dilution of 1:250. After 24 h, the cells were incubated overnight with corresponding secondary antibody dilutions, a donkey anti-sheep Alexa Fluor 647 (Sigma-Aldrich), a goat anti-rabbit Alexa Fluor 488 (Thermo Fisher Scientific, Invitrogen) and a donkey anti-goat Alexa Fluor 488 (Thermo Fisher Scientific, Invitrogen), all at a dilution of 1:250 in a 0.1 % BSA solution. Together with the secondary antibodies, the cell nuclei were stained with diamidino-2-phenylindole dihydrochloride (DAPI; Sigma-Aldrich) at a dilution of 1:200 in a 0.1 % BSA solution. The samples were imaged using confocal laser scanning fluorescence microscopy (TCS SP8 STED, Leica Microsystems), by acquiring series/z-stacks of 2 μm thick slices.

Quantification of the coverage of the microwells by the HUVECs was performed using CellProfiler (https://cellprofiler.org/). The coverage was calculated as percentage of the microwell area. To this, we first measured the (maximum) projection area of the circular microwells at their upper rim in pixels. Then, the area covered by the HUVECs was measured as the area of their CD31 fluorescence staining in pixels. The measurements were conducted in 4 microwells.

Quantification of the expression of selected angiogenesis markers by the HUVECs was performed using ImageJ (https://imagej.nih.gov/ij/) by identifying the area in μm^2^ corresponding to the fluorescence signal of each marker. The expression was calculated per cell, by normalizing for the number of counted nuclei. For the quantification of CD31 and laminin, 4 spheroids were imaged, and for podocalyxin, 2 spheroids. The quantification of the number of sprouts and the total sprouting length was performed with 4 spheroids using ImageJ, by a plugin named “Angiogenesis Analyzer” [42] and using the CD31 images as image source.

### 2.4 hDLEC culture on the microwell arrays and sprouting characterization

On the day of the experiment, microwell arrays were coated with a gelatin (Sigma-Aldrich) solution and rinsed in DPBS (Sigma-Aldrich), as described in section 2.3. hDLECs were trypsinized, re-suspended in fresh EMV2 (PromoCell) and counted. The hDLECs at passage 7 were seeded onto the microwell arrays at a density of 10,0000 cells per array and kept in culture at 37 °C and 5 % CO_2_ for 1 day, after which the upside-down porous microwells were flipped back. Then, the EMV2 was substituted with 50 % v/v Matrigel (Corning) in EMV2. The Matrigel in EMV2 was allowed to gel at 37 °C for 30 min, at which point 100 μL of EMV2 supplemented with VEGF-C (PeproTech; 100 ng/mL) [43] was dispensed on top of the Matrigel. The VEGF-C-supplemented EMV2 was refreshed every day for the whole duration of the culture.

The hDLECs were cultured on microwell arrays with diluted Matrigel to evaluate their ability to promote new vessel-like structure formation and sprouting after 1 and 3 days of culture by immunostaining of relevant endothelial markers. To this end, the hDLECs were fixed, permeabilized and blocked as described in section 2.3. Subsequently, the cells were incubated with a 0.1 % BSA (VWR) solution containing a dilution of the primary antibodies. For this, the following antibodies were used: A sheep anti-CD31/PECAM-1 (R&D Systems) and a goat anti-podocalyxin (R&D Systems), both at a dilution of 1:250. After 24 h, the cells were incubated overnight with corresponding secondary antibody dilutions, a donkey anti-sheep Alexa Fluor 647 (Sigma-Aldrich) and a donkey anti-goat Alexa Fluor 488 (Thermo Fisher Scientific, Invitrogen), both at a dilution of 1:250 in a 0.1 % BSA solution. Together with the secondary antibodies, the cell nuclei were stained with DAPI as described in section 2.3. The samples were imaged using confocal laser scanning fluorescence microscopy (TCS SP8 STED, Leica Microsystems) by acquiring z-stacks of 2 μm thick slices.

### 2.5 HUVEC luminal structure assay

The ability of the HUVECs for self-assembling into luminal structures within the microwells was assessed by performing a diffusion assay. The assay was carried out on HUVECs cultured on microwell arrays made from the film with 0.8 μm pore diameter, to exploit the sprouts formed through the pores as communication channels between the two compartments separated by the porous microwell wall. Briefly, HUVECs were seeded onto gelatin (Sigma-Aldrich) pre-coated porous microwells, as described in section 2.3. The cells were kept in culture in EGM2 (PromoCell) for 4 days. At day 4, the HUVEC-covered microwell arrays were removed from the 96-well plate, mounted in CellCrown 96-well plate inserts (Scaffdex) and moved to a new 96-well plate into which before per well 50 μL of 50 % Matrigel (Corning) in EGM2 were dispensed. Subsequently, the microwell arrays were incubated for 20 min at 37 °C and 5 % CO_2_ to let the Matrigel completely solidify. At this point, a solution of 10 μg mL^−1^ of fluorescein-isothiocyanate (FITC)-dextran (Sigma-Aldrich) with an average molecular weight of 2000 kDa in EGM2 was dispensed on top of the microwell arrays in the culture inserts. Images were taken 5, 10 and 20 min after the FITC-dextran administration using a fluorescence microscope (ECLIPSE Ti; Nikon Instruments). Medium was refreshed and new FITC-dextran added every 24 h. After 3 days of culture, the cells were fixed and permeabilized and an unspecific binding blocking step was performed as described in section 2.3. Then, the cell nuclei and F-actin were stained with DAPI (Sigma-Aldrich) and Alexa Fluor 568 phalloidin (Thermo Fisher Scientific, Invitrogen), respectively, both at a dilution of 1:200, overnight. Images were taken using a confocal laser scanning fluorescence microscope (TCS SP8 STED; Leica Microsystems), by acquiring z-stacks of 2 μm thick slices.

### 2.6 Aggregation and culture of hMSC spheroids in the microwell arrays

The day before the experiment, non-porous 289-microwell arrays were coated with 250 μL of 1 % w/v Pluronic F108 (Sigma-Aldrich) in sterile water (Milli-Q; Millipore) and incubated overnight at 37 °C to create a low-adherent surface in the microwells. Afterwards, microwell arrays were rinsed twice in DPBS (Sigma-Aldrich). hMSCs at passage 4 were trypsinized and re-suspended in fresh growth medium and seeded onto the microwell arrays at a density of approximately 5000 cells per microwell and incubated at 37 °C and 5 % CO_2_ for at least 2 h. After this, the medium was carefully refreshed to remove single cells that did not sink into the microwells and the cells were cultured for 48 h to allow full spheroid formation and compaction.

### 2.7 Aggregation and culture of MG-63 spheroids in the microwell arrays

The day before the experiment, non-porous 289-microwell arrays were coated with 250 μL of 1 % w/v solution of Pluronic F108 (Sigma-Aldrich) in sterile water (Milli-Q; Millipore) and incubated overnight at 37 °C. Subsequently, microwell arrays were rinsed twice in DPBS (Sigma-Aldrich). MG-63 cells at passage 98 were trypsinized, re-suspended in fresh growth medium (see section 2.2) and seeded onto the microwell arrays at a density of approximately 15,000 cells per microwell and incubated at 37 °C and 5 % CO_2_ for at least 3 h. After this, the medium was carefully refreshed and the cells were cultured for 48 h to allow for a full spheroid formation and compaction.

### 2.8 hMSC spheroid vascularization

A co-culture of hMSC spheroids and HUVECs was performed on the microwell arrays to model spheroid vascularization *in vitro*. On the day of the experiment, pre-formed hMSC spheroids were retrieved from the 289-microwell arrays using a P200 pipette. The collected spheroids were re-suspended in 50 % Matrigel in a co-culture medium, which was composed of 50 % hMSC growth medium and 50 % EGM2 (PromoCell), and dispensed onto the HUVEC-seeded porous and non-porous microwell arrays and incubated at 37 °C and 5 % CO_2_ for 1 and 3 days. After that, the cells were fixed and permeabilized and an unspecific binding blocking step was performed as described in section 2.3. Stainings targeting CD31 on the HUVEC cell membrane, laminin and podocalyxin were performed as described in section 2.3. Image acquisition through a confocal laser scanning fluorescence microscope (TCS SP8 STED; Leica Microsystems) provided z-stacks of slices of 3 μm thickness. What concerns the culture durations, we investigated also longer durations of up to 7 days (Supplementary Fig. 1). However, after 5 days of culture, signs of a disintegration of the hMSC spheroids were found. We considered the integrity of the spheroids an essential feature of the model and an important aspect to maintain throughout the experiments though. A prolonged culture duration of 5 and more days for spheroids from hMSCs might therefore need an optimization of the matrix for HUVEC sprouting and spheroid embedding.

### 2.9 MG-63 spheroid vascularization

Similarly to what was described in section 2.9 for hMSC spheroids*,* a co-culture of MG-63 spheroids and HUVECs was performed on the microwell arrays to model tumor spheroid vascularization *in vitro*. On the day of the experiment, pre-formed MG-63 spheroids were retrieved from the 289-microwell arrays using a P1000 pipette. The collected spheroids were re-suspended in 50 % Matrigel in a co-culture medium, which was composed of 50 % growth medium and 50 % EGM2 (PromoCell), and dispensed onto the HUVEC-seeded porous and non-porous microwell arrays and incubated at 37 °C and 5 % CO_2_ for 1 and 3 days. Afterwards, the cells were fixed and permeabilized and an unspecific binding blocking step was performed as described in section 2.3. A triple staining targeting CD31 on the HUVEC cell membrane, laminin and podocalyxin was performed as described in section 2.3. Images acquisition through a confocal laser scanning fluorescence microscope (TCS SP8 STED; Leica Microsystems) provided z-stacks of slices of 3 μm thickness.

### 2.10 Transmission electron microscopy analysis of vascularized hMSC spheroids

After 1 and 3 days in culture on the microwell arrays, the HUVEC-vascularized hMSC spheroids were treated for transmission electron microscopy (TEM) imaging following the steps of Bonanini et al. [44]. Briefly, the samples were fixed with a solution of 1.6 % glutaraldehyde (Merck) in 0.1M phosphate buffer (Merck) for 24 h at 4 °C, which also dissolved the Matrigel. Once the Matrigel was completely dissolved, the spheroids were removed from the microwell arrays and transferred to a wash buffer (0.1 M cacodylate; Acros). Afterwards, the samples underwent an additional fixation step with a solution of 1 % osmium tetroxide (Ted Pella) and 1.5 % potassium ferricyanide (Merck) in the wash buffer for 1 h at 4 °C in the dark. Then, the samples were dehydrated by using an ethanol solution series ranging from 70 % to 100 % ethanol in sterile water (Milli-Q; Millipore) and infiltrated with Epon resin (Ladd Research) for 2 days. Next, the samples were embedded in Epon resin and allowed to polymerize for 2 days at 60 °C. 70 nm-thick sections were cut with an ultramicrotome (Ultracut UCT; Leica) and mounted on Formvar-coated copper grids (Electron Microscopy Sciences). The sections were stained with 2 % uranyl acetate (SPI Supplies) in water and lead citrate (Merck), and imaged using a Tecnai T12 electron microscope and an Eagle 4 k∗4 k CCD camera (ThermoFisher Scientific).

### 2.11 Statistical analysis

All experiments were performed in each case in/with 4 microwells and/or spheroids (2 microwells and/or spheroids per array from in each case 2 microwell arrays), unless stated otherwise. The quantified results were presented as mean ± standard deviation and statistical analyzes were performed in Prism 8 (GraphPad) using a two-way analysis of variance (ANOVA) followed by a Tukey's post-hoc test for multiple comparisons. ‘∗’, ‘∗∗’ and ‘∗∗∗’ indicate statistically significant differences with p-values smaller than 0.05, 0.01 and 0.001, respectively.

## 3 Results and discussion

### 3.1 Microwell array fabrication and characterization

**Fig. 2:**
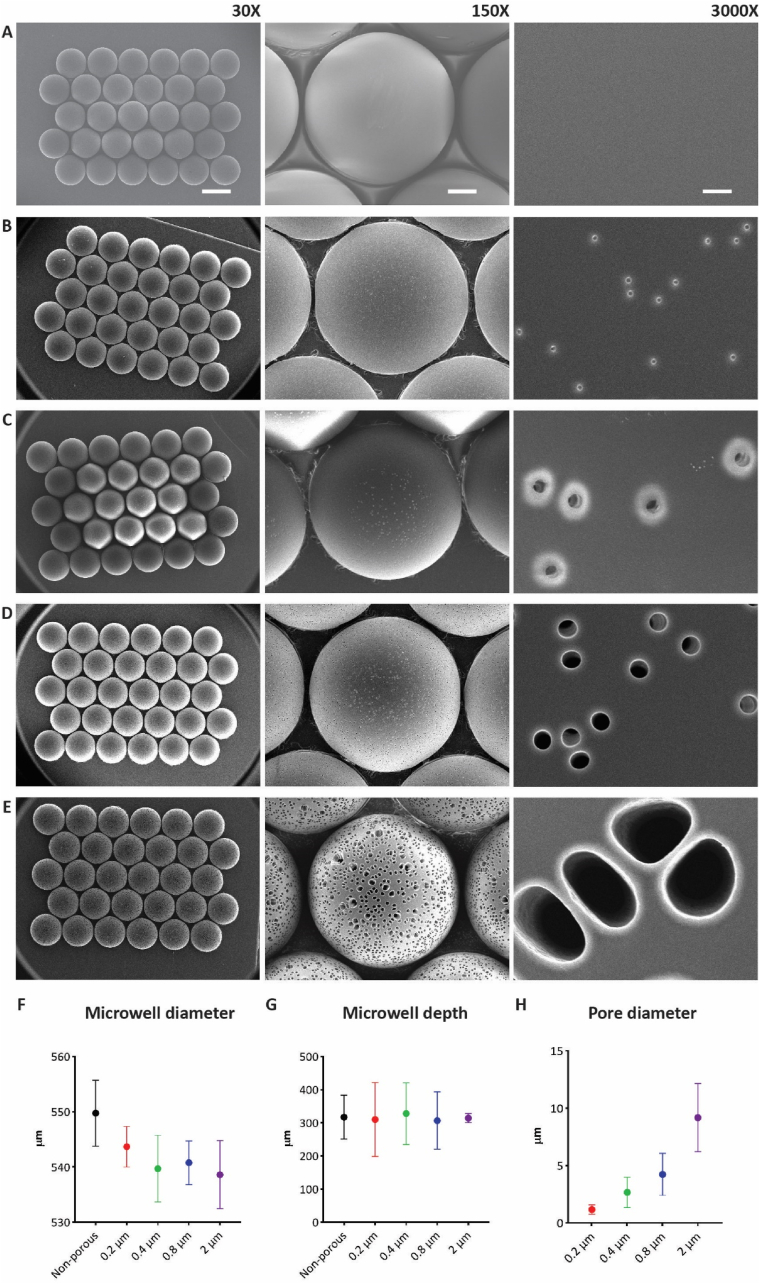
**Visual inspection and geometrical characterization of the microwell arrays:** (A–E) Back/bottom view SEM images of 30-microwell arrays made of (A) non-porous PC film and porous microwell arrays made of porous PC films with nominal pore diameters of (B) 0.2, (C) 0.4, (D) 0.8 and (E) 2 μm. Images were acquired at magnifications of 30×, 150× and 3000×. The scale bars represent 500, 100 and 5 μm, respectively, and apply to all images of the respective magnification column. (F–H) Characterization of the microwells' geometrical parameters in dependence of the original films used for their fabrication by optical profilometry including (F) (maximum) average outer microwell diameter, (G) (maximum) average microwell depth/height and (H) (maximum) average pore diameter.

For the present study, a non-porous and a porous microwell setup were developed to study vascularization *in vitro*. Non-porous and porous microwell arrays were fabricated via gas-assisted microthermoforming of thin PC films, as previously described by our group [29,41]. Non-porous microwell arrays (Fig. 2A) were obtained by dense PC films. For the porous arrays (Fig. 2B–E), four ion track-etched PC films with different pores diameters, nominally with 0.2, 0.4, 0.8 and 2 μm, were selected. The thermoforming of already (commercially available) track-etched films is a novel process recently described by Baptista et al. [41]. Before, the production of porous thermoformed microwells in our studies consisted of first gas-assisted thermoforming of (commercial) heavy ion-irradiated but not yet etched and therefore still dense films and then wet etching to open the pores [38,45]. Thermoforming of already porous films speeds up the fabrication process as it makes the final wet etching superfluous.

A qualitative morphological assessment of the microwell arrays showed successful microwell fabrication outcomes in terms of consistency of geometrical features, such as outer diameter and depth of the microwells, across the different non-porous and porous microwells. This indicates that the initial characteristics of the films did not visibly affect the forming process. Quantitative measurements of the microwells' (maximum) outer diameter and depth confirmed the qualitative observations (Fig. 2F and G, respectively). The (maximum) outer microwell diameter, measured at the upper rim of the microwells, and (maximum) microwell depth/height, measured at the apex of the microwells from their backside, was on average slightly less than 550 and a little bit more than 300 μm, respectively.

As expected, the average diameter of the pores at the apex changed during thermoforming (Fig. 2H). Pore sizes of 0.2, 0.4, 0.8 and 2 μm became 1.17 ± 0.41, 2.67 ± 1.32, 4.24 ± 1.83 and 9.19 ± 2.97 μm, respectively. The greatest stretch factor was found for the 0.4 μm-pore films, which were enlarged (up to) 6.68-fold. The smallest stretch factor was found for the 2 μm-pore films, which were (up to) 4.59-fold enlarged. However, in the latter case, an extensive fusion of pores was observed (Fig. 2E, middle column), suggesting this film as rather unsuitable for the free-forming of deep defined-porous structures, including the microwells of this study.

### 3.2 Microwell arrays as 3D *in vitro* endothelial sprouting platforms

HUVECs were cultured on the microwell arrays to investigate cell behaviors such as cell attachment, survival and sprouting for the proposed culture setup. The cells were cultured in EGM2 for 4 days, after which the medium was switched to a 3D matrix composed of 50 % Matrigel in EGM2 where the cells were cultured for another day.

**Fig. 3:**
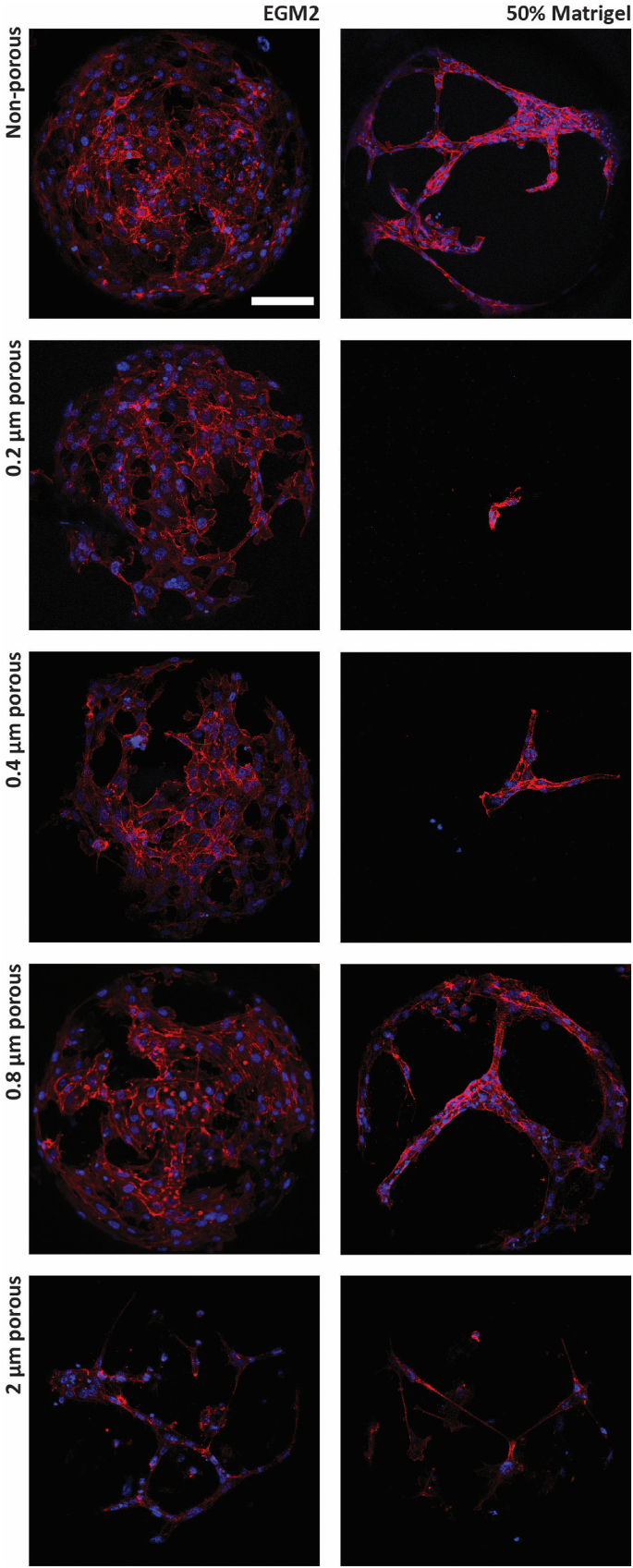
**Culture of HUVECs onthemicrowell arrays:** Maximum intensity projections of confocal fluorescence microscopy images of HUVECs on microwell arrays after 4 days of culture in EGM2 (left column) and 1 additional day in 50 % Matrigel in EGM2 (right column) for the different original films used for the fabrication of the microwells. The cells were stained with DAPI (in blue) and phalloidin (in red) to label cell nuclei and the F-actin, respectively. The scale bar represents 100 μm and applies to all images.

After 4 days of culture in EGM2, the HUVECs adhered on the surfaces of the non-porous microwells and the ones made from the 0.2, 0.4, 0.8 and 2 μm-pore films, with varying degrees of surface coverage (Fig. 3, left column). Overall, HUVECs on the surfaces of non-porous microwells showed the highest surface coverage, with very small gaps left between the cells. HUVECs on the porous microwell surfaces, with the exception of the ones from the 2 μm-film, did not show any remarkable differences in terms of microwell colonization. The cells appeared homogeneously dispersed over the microwell surface. HUVECs on the surfaces of microwells from the 2 μm-film showed a sparser colonization in comparison to the other (porous) films (Fig. 3, bottom row, left column). What was just described qualitatively was confirmed via image analysis of the HUVECs' surface coverage using CellProfiler (Supplementary Fig. 2). An explanation for the differences in the coverage results may lay in the high porosity of the microwells from the 2 μm-pore films, which had pores enlarged up to an average diameter of around 9.2 μm (Fig. 2H). As for most human cells, adhering HUVECs can measure tens of micrometers in diameter, depending on the investigated substrate [46], but they are smaller when in suspension, for example, during the seeding process. Thus, apart from a potential challenge for the cells to adhere on the highly porous and rough substrate, another possible reason for the lower numbers of HUVECs attaching on the surfaces of microwells from the 2 μm-film might have been that during seeding cells sank through the pores and landed onto the bottom of the well plate underneath.

What concerns the subsequent culture in diluted Matrigel (Fig. 3, right column), HUVECs on non-porous microwell arrays rapidly transitioned from adhering on the surface of the microwells to sprouting into a 3D network suspended into the Matrigel (Fig. 3, as labeled, and Supplementary Video 1A). The HUVECs cultured on microwell arrays from the 0.8 μm-film showed a similar behavior, by compacting into tubular-like structures in the microwells (Fig. 3, as labeled, and Supplementary Video 1B). For the other conditions, very limited amounts of sprouting was visible (Fig. 3, as labeled, and Supplementary Videos 1C–E). For microwell arrays from the 0.2 μm-film, the HUVECs might have been too large to elongate and sprout through the stretched and widened pores of the microwells. Similarly, also the HUVECs seeded onto microwell arrays from the 0.4 μm-film did not exhibit a bigger amount of sprouting through the pores. Nevertheless, the few present sprouts appeared structured and organized. Although to our knowledge the minimal diameter of HUVEC-formed luminal structures *in vitro* has not been defined, previous studies reported the inner diameter of capillaries formed by HUVECs to be inferior to 10 μm, but very rarely smaller than 3 μm [47]. For microwell arrays from the 2 μm-film, very little sprouting was visible but in this case the reason was probably the considerably lower number of cells that was available for the sprouting process. In fact, the on average around 9.2 μm in diameter after microthermoforming is very close to the 10 μm pore diameter previously described as ideal to promote vascularization through (porous) membranes *in vivo* [48].

The microwell arrays from the 0.8 μm-film outperformed the other porous microwell arrays in terms of HUVECs' adhesion and sprouting behavior in Matrigel. Therefore, together with the non-porous ones, the microwell arrays from the 0.8 μm-film were selected to further investigate vascularization mechanisms on the platform and from this point on referred to as “porous microwell(array)s”.

Supplementary data related to this article can be found online at https://doi.org/10.1016/j.mtbio.2024.101260

The following are the Supplementary data related to this article:

Multimedia component 2 Multimedia component 2

Multimedia component 3 Multimedia component 3

Multimedia component 4 Multimedia component 4

Multimedia component 5 Multimedia component 5

Multimedia component 6 Multimedia component 6

### 3.3 Characterization of HUVEC sprouting on the microwell arrays

After selecting the optimal pore diameter for the porous microwells, a characterization of the 3D culture and sprouting behavior of the HUVECs in both non-porous and porous microwells was performed via immunostaining of relevant vascular endothelial markers, fluorescence imaging and image analysis. For all readouts, after a growth phase in EGM2 for 4 days, HUVECs were cultured in 50 % Matrigel in EGM2 for another 1 or 3 days. As vascular markers, CD31, a typical marker expressed by endothelial cells [49], laminin (α1), a vascular basement membrane protein [50], and podocalyxin, an apical (transmembrane) protein in luminal endothelial structures [51], were selected.

**Fig. 4:**
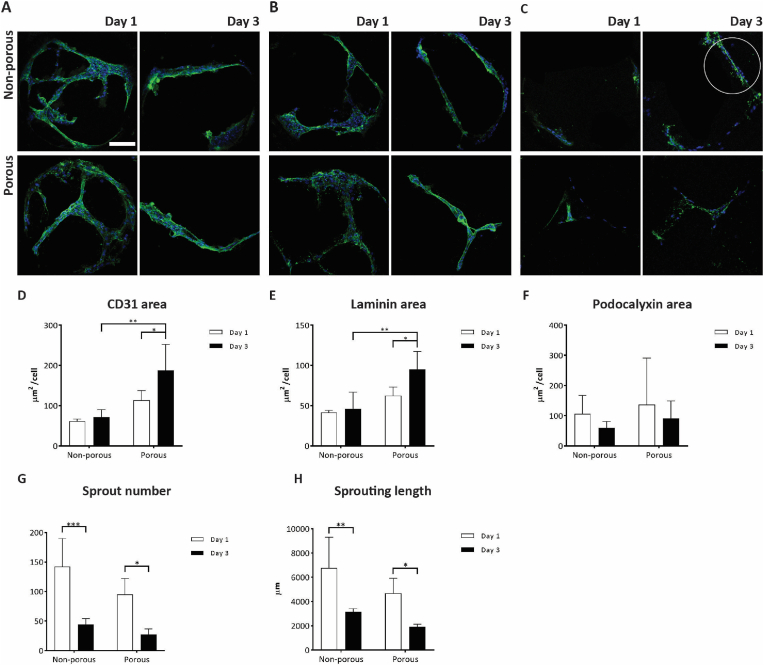
**Visualization and quantification of the expression of vascular markers by HUVECs cultured on non-porous and porous microwell arrays after 1 and 3 days of culture in 50 % Matrigel in EGM2:** (A–C) Maximum intensity projection of confocal fluorescence microscopy images of HUVECs in microwell arrays. (A) Cell nuclei and CD31 are stained in blue and green, respectively. (B) Cell nuclei and laminin are stained in blue and green, respectively. (C) Cell nuclei and podocalyxin are stained in blue and green, respectively. The scale bar represents 100 μm and applies to all images of the subfigures (A)–(C). (D–F) Quantification of vascular markers in terms of area coverage by image analysis in ImageJ for (D) CD31, (E) laminin and (F) podocalyxin. (G and H) Geometrical characterization of the sprouting network in terms of (G) sprout number and (H) sprouting length in ImageJ plugin Angiogenesis Analyzer.

From qualitative observations, it could be seen that HUVECs in non-porous and porous microwells expressed abundantly CD31 and laminin (Fig. 4A and B, respectively). Partially co-localized, these two proteins marked the whole cell membrane [52] and the basement membrane [53], respectively. HUVECs seemed to express podocalyxin at the apical side of the sprouted structures, similarly to what is reported in literature [54,55], in non-porous microwells after 3 days in culture (Fig. 4C, as labeled, in the white circle, and Supplementary Videos 2 and 3). However, after 1 day in culture for both types of microwells as well as at both time points for porous microwells, podocalyxin was only faintly detectable and without a clear association to a lumen (Fig. 4C, left column and bottom row). This might indicate that in this case the vessel-like networks are not yet fully mature(d) structures. A further maturation of the vessels might be helped by tuning their environment through the addition of other extra cellular matrix (ECM) protein components and stromal cells, such as fibroblasts, to it.

Supplementary data related to this article can be found online at https://doi.org/10.1016/j.mtbio.2024.101260

The following are the Supplementary data related to this article:

Multimedia component 7 Multimedia component 7

Multimedia component 8 Multimedia component 8

Multimedia component 9 Multimedia component 9

Multimedia component 10 Multimedia component 10

When quantifying the endothelial vascular markers on both non-porous and porous microwell arrays by image analysis (Fig. 4D), the expression of CD31 per cell in non-porous microwells showed comparable values after 1 and 3 days in culture. For porous microwells, instead, a significant increase of CD31 expression from day 1 to day 3 was identified. When comparing the two microwell array types, although the quantified CD31 after 1 day was comparable, after 3 days, cells in porous microwells expressed significantly more CD31 than in non-porous ones. The quantification of the laminin-covered area per cell showed a similar trend, although the amount of this protein was lower than that of CD31 (Fig. 4E). These results suggest two important considerations for the microwell-based vascularization setup. On the one hand, the HUVECs seeded onto porous microwell arrays show a delay during the formation of 3D vascular networks within the microwells, in comparison to the HUVECs in non-porous microwells. A probable cause for this could be the additional distance the HUVECs have to travel through the pores to the inner side of the microwells. One the other hand, after 3 days, HUVECs in porous microwells outperform HUVECs in non-porous ones what concerns their expression of CD31 and laminin. CD31 and laminin can give an indication of the extension of the cell border [56] and of the cell activity regarding sprouting and tube formation [57], respectively. Hence, these results may indicate that HUVECs seeded onto porous microwell arrays formed more structurally solid vascular networks in comparison to non-porous ones, although in a longer period of time. The area per cell, associated to the podocalyxin marker did not show any remarkable difference between the conditions and time points (Fig. 4F).

To have a clearer understanding of the spatial organization of the HUVEC network within the microwells, we analyzed the morphology of the sprouting by quantifying the total number of sprouts per microwell and their total length in μm (Fig. 4G and H, respectively). This analysis was performed by processing the CD31 fluorescence images using the ImageJ plugin Angiogenesis Analyzer [42]. The steps of an exemplary pipeline used for this can be followed in Supplementary Fig. 3. For the quantification, the total number of vascular segments and branches was considered, as well as their total length when all combined. As indicated in Supplementary Fig. 3C and following the guidelines of the plugin authors, a “branch” represents a sprout that protrudes from a junction point, also known as a “node”, toward an empty space without connecting or crossing any other sprouts. On the other hand, a “segment” extends between two nodes. We defined and named the combination of branches and segments as “sprouts” and the total sum of their lengths as “sprouting length”, as both was considered relevant for understanding the sprouting behavior and vascular network formation of HUVECs in the microwell arrays. The quantification of these two parameters showed outcomes different to what was previously analyzed for the endothelial markers, particularly concerning the CD31 and laminin results. There were significantly less sprouts after 3 days in culture in comparison to 1 day. The total sprouting length also decreased from day 1 to day 3 of the culture. The progressive loss of structural integrity of HUVEC sprouts in Matrigel was similarly reported in connection with a bioprinting approach, where these cells rapidly disassembled their previously formed sprouting structures from day 1 to day 2 [58]. The authors of this study successfully employed a mixture of Matrigel and thrombin to enhance HUVECs confinement and adhesion. Therefore, the HUVEC's sprouting behavior in the non-porous and porous microwells was possibly also due to the choice of Matrigel (alone). *In vivo*, the formation of networks of endothelial cells is supported by other cells, such as stromal fibroblasts, and mural vascular smooth muscle cells and pericytes [59,60]. The absence of these cells in our system might be one explanation for the reduction of the sprouts' number and overall length observed in our system over time. Stromal fibroblasts play a crucial and supportive role in endothelial network formation and vascularization by stimulating the proliferation, differentiation and lumen formation of endothelial cells through the secreting pro-angiogenic factors, such as VEGF, fibroblast growth factor (FGF) and platelet-derived growth factor (PDGF) [[61], [62], [63]]. They also contribute to ECM remodeling and regulating angiogenesis by secreting matrix metalloproteinase enzymes [64]. Furthermore, stromal fibroblasts support the stabilization of vascular/endothelial networks by producing matrix [59,61,65] and involvement in pericyte recruitment and formation [60,65]. Therefore, co-culturing endothelial cells with stromal cells is a promising strategy for generating more mature vascular networks and promoting angiogenesis. The platform described in this study provides a compartmentalized environment that can facilitate the co-culture of multiple cell types. For example, on our platform, stromal fibroblasts could be cultured in the hydrogel along with the endothelial cells and spheroids. This configuration could be used to study the effects of stromal fibroblasts on the behavior of the endothelial cells, the formation of vascular networks and the vascularization of cellular aggregates.

The optimized microwell-based platform appears suitable to support HUVEC growth and sprouting *in vitro*. Engineering of functional tissue and organ models or equivalents often requires the integration of artificial blood vascular networks in the same. More and more efforts are also being directed toward integrating another vasculature, the lymphatic vasculature, into tissue models, such as corresponding on-chip platforms [66]. The lymphatic system is a blind-ended set of vessels that collect the lymph from the tissue interstitial space to bring it to the lymph nodes. Modeling and studying of the lymphatic system has also gained interest because of its involvement in several disease mechanisms [67,68]. As a proof of concept, in the current study, we showed the feasibility of hDLECs to grow and form lymphatic sprouts within the microwell-based vascularization platform (Supplementary Fig. 4). Lymphangiogenesis occurs upon the presence of VEGF-C, toward which hDLECs form new sprouts [69]. hDLECs expressed podocalyxin and CD31, as expected from previous studies [70,71]. Through the selected lymphatic markers, we observed the formation of hDLEC sprouts within non-porous microwells mostly already after 1 day (Supplementary Video 4A). The quick sprouting of hDLECs has been previously reported in literature on a different *in vitro* platform [43]. The poor maintenance of the lymph vessels' shape and function within 3 days (Supplementary Video 4B) may be attributed to the fast dissolution of the gradient of VEGF-C [68] within the microwell as the Matrigel may have saturated. For porous microwells, no hDLEC sprouting process was observed over the culture time. Although it is still unclear why this is the case and in this regard further research is needed, it was previously reported that hDLECs interacting with a porous substrate may undergo cytoskeletal re-arrangement and therefore experience disturbances of their sensing apparatus [43]. This might have been also the reason for the absence of sprouting through the walls of the porous microwells.

Additionally to the immunostaining of podocalyxin, to effectively assess the presence of a lumen of the HUVEC sprouts, FITC-dextran was allowed to diffuse through formed structures as described in the materials and methods section/section 2. Here, a FITC-dextran of a molecular weight of 2000 kDa was selected as this could travel through microwell pores and subsequently connected sprouts but was unlikely to permeate through the wall of sprouts [72]. Already after 5 min, it was possible to identify some FITC-dextran trapped within the endothelial structures (Supplementary Fig. 5A), which can be considered an indication of the presence of a lumen within the sprouts [73]. The fluorescence signal faded rapidly over time and already after 10 and 20 min appeared grainy and spotted (Supplementary Fig. 5B and C, respectively). After 3 days in culture and a staining procedure, the sprouts still presented some accumulation of green dots within their volume (Supplementary Fig. 5D). These finding were in line with results from similar *in vitro* angiogenesis platforms [44,74] and of fundamental importance as the presence of a non-leaking lumen of a sprout is an essential characteristic of its maturation, integrity and function as perfusion systems [74]. However, it is worth mentioning that the intentional use of dextran with such a high molecular weight as the one chosen hindered insights on the permeability of the vessels, the characterization of which is also important to understand the vessels' maturation as cell-cell junctions form [74]. Nevertheless, together with the podocalyxin data, these results were considered sufficient to move to the next step, the investigation of the microwell platform's capacity for the vascularization of 3D cell aggregates.

### 3.4 HUVEC sprouting on the microwell arrays as vascularization platforms for cell spheroids

**Fig. 5:**
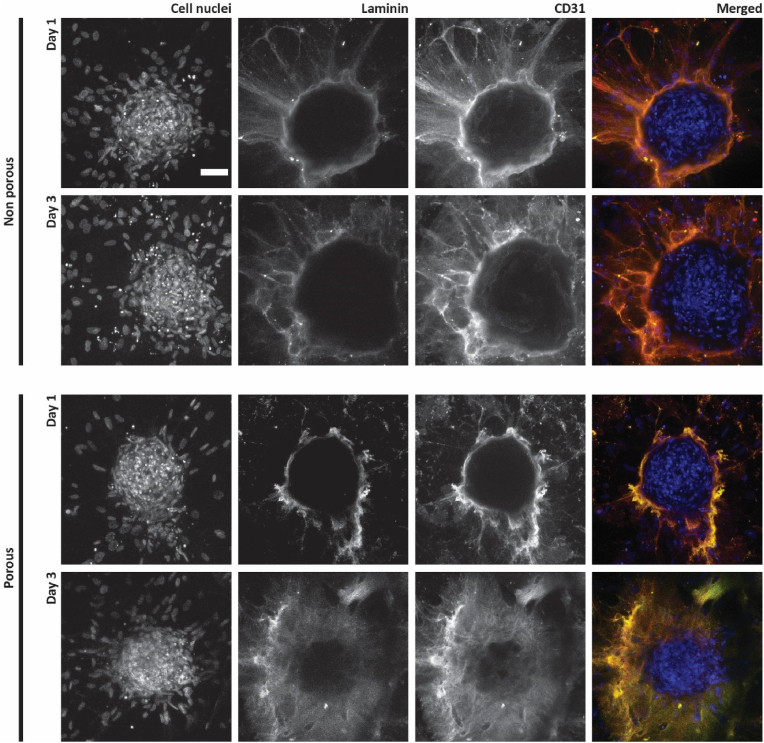
**Assessment of hMSC spheroid vascularization by laminin staining:** Maximum intensity projections of confocal fluorescence microscopy images of co-cultures of hMSC spheroids and HUVECs on non-porous and porous microwell arrays after 1 and 3 days in 50 % Matrigel in co-culture medium. Cell nuclei, laminin and CD31 are stained in blue, green and red, respectively (shown only in the merged images). The scale bar represents 50 μm and applies to all images.

**Fig. 6:**
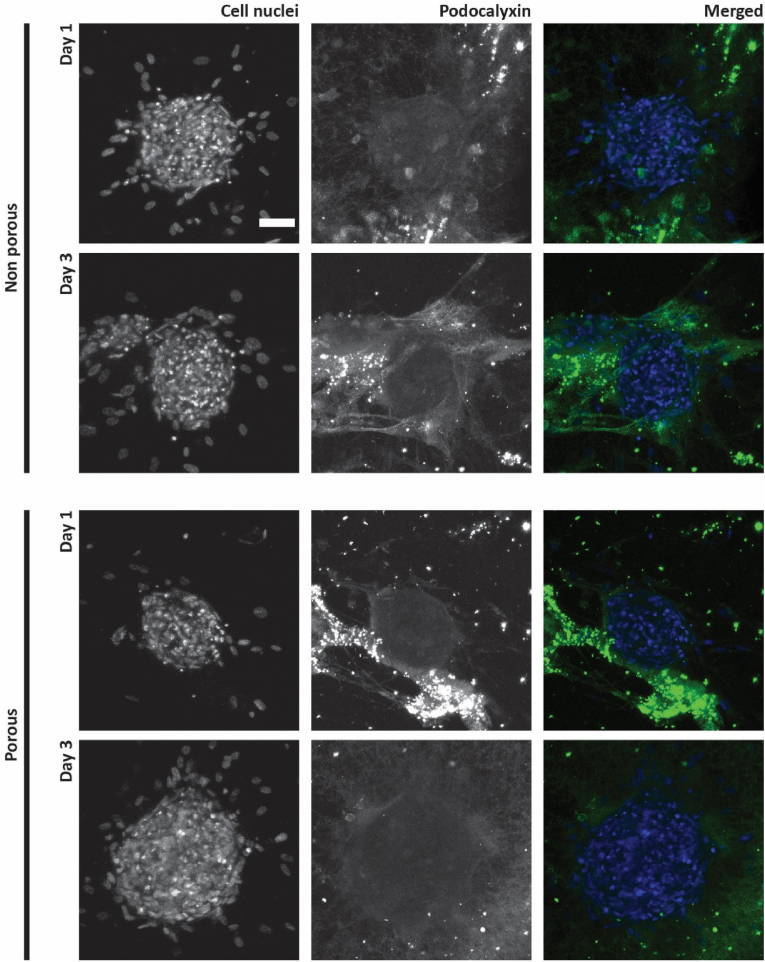
**Assessment of hMSC spheroid vascularization by podocalyxin staining:** Maximum intensity projections of confocal fluorescence microscopy images of co-cultures of hMSC spheroids and HUVECs on non-porous and porous microwell arrays after 1 and 3 days in 50 % Matrigel in co-culture medium. Cell nuclei and podocalyxin are stained in blue and in green, respectively (shown only in the merged images). The scale bar represents 50 μm and applies to all images.

To investigate the capability of the microwell arrays as platforms for the vascularization of 3D cell aggregates, hMSC spheroids were seeded onto non-porous and porous microwell arrays on which HUVECs have been pre-cultured. Before, hMSC spheroids were formed in a non-porous 289-microwell array (Supplementary Fig. 6A). After spontaneous aggregation and culture for in total 48 h (Supplementary Fig. 6B), the hMSC spheroids were transferred to the HUVEC-covered microwell arrays. At this time, the spheroids had an average diameter of 208.61 ± 23.28 μm (quantified from the uncropped image of Supplementary Fig. 6B), indicating a good size uniformity, as similarly found by us for (pure) hMSC spheroids in a previous study [29]. The co-culture was performed up to 1 and 3 days in 50 % Matrigel in co-culture medium, as described in section 2.8. To assess the HUVEC's behavior in terms of sprouting and expression of vascular markers, two different sets of immunostainings were performed, for laminin and CD31 (Fig. 5) and for podocalyxin (Fig. 6).

The formation of a vascular network sprouting from the microwell walls and reaching the hMSC spheroids occurred in both non-porous and porous microwell arrays (Fig. 5). After 1 day in culture, qualitative observations of CD31 and laminin expression suggested the HUVEC sprouting toward the hMSC spheroid being more abundant in non-porous microwells compared to porous ones. On the other hand, at day 3, the amount of HUVEC sprouting appeared comparable for the two different microwell types.

**Fig. 7:**
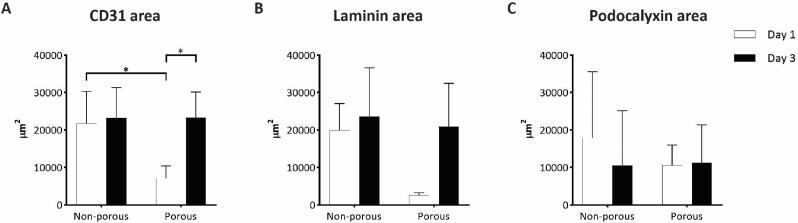
**Quantification of vascular markers of HUVECs after 1 and 3 days of co-culture with hMSC spheroids on non-porous and porous microwell arrays in 50 % Matrigel in co-culture medium:** Quantification of selected vascular markers including (A) CD31, (B) laminin, (C) podocalyxin in terms of coverage of the area of their expression by HUVECs in co-culture with hMSC spheroids quantified via image analysis in ImageJ.

Quantitative image analysis in ImageJ confirmed these observations, particularly when looking at the total CD31 area (Fig. 7A). This showed the CD31 expression being constant at the selected time points for co-cultures in non-porous microwell arrays, while increasing over time for the co-culture in porous microwell arrays. HUVECs in non-porous microwells seemed to immediately reach the hMSC spheroid to form an extended vascular network, the extension of which in terms of covered area of marker expression did not change over time. On the other hand, HUVECs on porous microwell arrays experienced a delay in reaching the hMSC spheroid and in forming a vascular network around it. The observation of delayed sprouting was in line with what was observed for the culture of HUVECs only. As discussed in section 3.3, the physical barrier generated by the microwell itself first needing to be overcome seems a plausible explanation for this outcome. However, the pores in the microwell walls define the locations from where only the HUVECs can start sprouting – which might open up possibilities for manipulating sprouting patterns –, concerning the porous microwell design from this study obviously without influencing the final amount of sprouts.

The quantification of laminin expression in terms of total area of expressed marker presented the same trend as the CD31 quantification (Fig. 7B). Although in this case, the quantified differences were not statistically significant.

Surprisingly, most of the HUVECs reaching the spheroids formed a layer on the external surface of the spheroids and only little penetration of a HUVEC network into the spheroids was visible, and the latter mostly only after 3 days in culture (Fig. 5). The compactness of the hMSC spheroids, which are known to shrink over time [29] and produce large amounts of ECM [75], may have hindered the HUVECs to penetrate the spheroids. In this regard, another aspect worth mentioning is the co-culture time. As reported in another study [44], the infiltration of HUVECs into hepatic spheroids occurred after 7 days. This suggests that with prolonged co-culture times a more extensive vasculature can be obtained inside the spheroid to complement to the outer vasculature hitherto achieved.

The expression of podocalyxin by the HUVECs when co-cultured with the hMSC spheroids was found with some minor qualitative discrepancies between conditions and time points, which however did not result in statistically significant quantitative differences (Fig. 6, Fig. 7). Podocalyxin plays an important role in maintaining vascular permeability as it regulates the endothelial barrier integrity [76]. Here, the investigation of podocalyxin expression by HUVECs when co-cultured with hMSC spheroids provided some insights on the maturity of the engineered vessel-like structures and thus on the performance of the microwell arrays as a vascularization platform for 3D cell aggregates. Although not fully characterized, it is possible to conclude that for both non-porous and porous microwell arrays an extensive vascular network formed within the microwells, connecting the spheroid outer layer to the surrounding environment. In addition, the expression of the selected vascular markers suggests a certain degree of functionality of the engineered vascular network.

The generation of functional vasculature is a critical aspect of many TE-based *in vitro* models and *in vivo* therapies. The size of engineered tissues *in vitro* cannot significantly exceed the distance found between two blood capillaries *in vivo* before it needs additional measures such as an engineered vasculature to sustain oxygen and nutrient supply [77]. In this context, there is a great need for innovative and versatile *in vitro* vascularization platforms. Here, non-porous and porous microwell arrays as co-culture platforms for spheroids and HUVEC-based vasculature demonstrated the potential to obtain vascularized spheroids *in vitro*. hMSC spheroids were chosen as a 3D model of a stromal tissue to evaluate the microwell arrays as effective *in vitro* vascularization platforms for 3D cell aggregates. MSCs are known to positively affect angiogenesis *in vitro* and *in vivo* [78]. Their secretome contains a plethora of angiogenic factors. Moreover, MSCs aggregated into spheroids express upregulated angiogenic markers in comparison to 2D cultures of MSCs [79]. To showcase the versatility of the platform, in addition to the hMSC spheroids, the vascularization of MG-63 spheroids was performed and characterized (Supplementary Fig. 7). Because of cell-specific interactions, a different cell source of the spheroids could be expected to have an effect on the vascularization process as it was observed so far, which was also the case. The MG-63 spheroids were also successfully vascularized by HUVEC sprouting within the microwell arrays, although overall less copious compared to the hMSC spheroids. A more pronounced inner vascularization of the MG-63 spheroids was observed when compared to the hMSC spheroids. This could be in accordance with the literature where it is discussed that a potential hypoxic core at the center of MG-63 (tumor) spheroids could have favored the secretion of VEGF, which helps to attract HUVECs to the spheroids, form vessel-like structures and organize vascular networks [80]. The same study also reports that the deposition of the tumor spheroids onto the HUVECs cultured as a monolayer first led to the death of the endothelial cells in direct contact with the tumor cells. This might explain the visibly lower number of HUVECs in the co-culture with MG-63 spheroids in comparison with the hMSC spheroids. In our results, this was particularly evident for co-cultures on non-porous microwell arrays, where the HUVECs and the MG-63 spheroids shared the same microwell space, probably partly in direct contact. This is in contrast to porous microwells, where the initial protective separation provided by the microwell walls might have allowed a higher amount of HUVECs to vascularize the MG-63 spheroid.

Taken together, the outcomes of HUVEC-based vascularization of hMSC and MG-63 spheroids in microwell arrays differed substantially. This suggests that the capacity of the designed co-culture approach to display different cell interactions occurring during vascularization processes of 3D cell aggregates.

Commonly, microwell-based vascularization of spheroids relies on the spatial confinement of cells [35] or pre-formed spheroids [34]. These methods, which generally make use of microwells casted from/in agarose, have demonstrated to be a good option for fabricating pre-vascularized spheroids for applications such as bioprinting and bottom-up TE. In comparison to that, our approach proposes a more active role of the microwell wall in the spheroid's vascularization process. The use of porous microwells as a substrate for the vascular bed showed a slower HUVEC sprouting and interaction with the hMSC spheroid, which significantly increased from day 1 to day 3 of the observation. On the other hand, non-porous microwells showed a high amount of vascular sprouts from day 1 in culture, with no changes over time. This indicates that the physical and chemical properties of the microwell array, i.e., of the micropores and the (for example, gelatin) microwell coating, respectively, can regulate the outcomes of the spheroid vascularization. The modulation of such properties may be useful to tailor the vascularization for different tissue types, for example, by adjusting parameters such as pore diameter, pore density and film thickness, the latter being identical to the pore length, depending to the microanatomy of the targeted tissue.

### 3.5 TEM analysis of hMSC spheroid vascularization

**Fig. 8:**
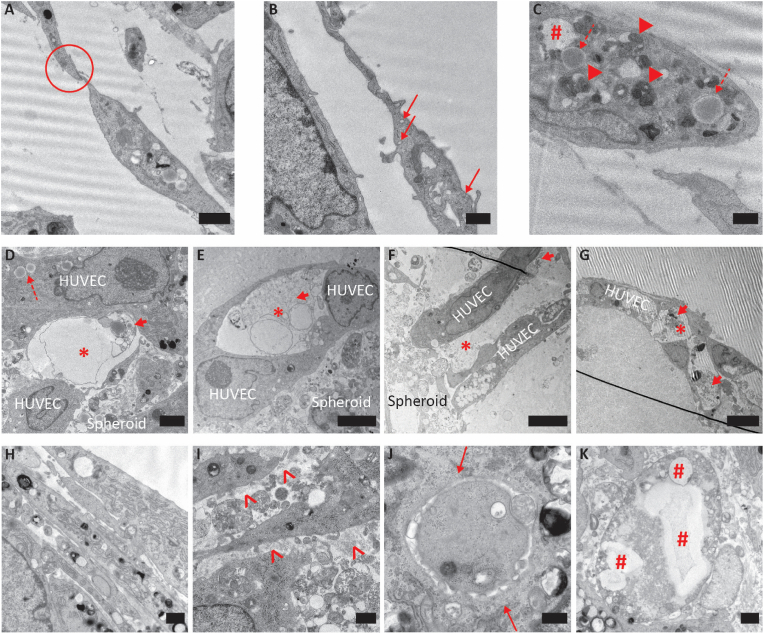
**TEM of HUVEC-vascularized hMSC spheroids after 1 and 3 days of culture on non-porous and porous microwell arrays in 50 % Matrigel in co-culture medium:** (A–G) Vascularized spheroids on non-porous microwell arrays after 3 days of culture. (A) Formation of TJs, indicated by the red circle. The scale bar represents 2 μm. (B) Presence of PVs, indicated by the red thin arrows. The scale bar represents 1 μm. (C) Presence of autophagosomes, indicated by the red arrowheads, round or electron-dense WPBs, indicated by the red dashed arrows, and secretory pods, indicated by the red hashtag. The scale bar represents 1 μm. (D–G) Evidences of formation of HUVEC-lined lumen-like structures, indicated by the red asterisks. (D) Lumen-like structure inside an hMSC spheroid. The scale bar represents 2 μm. (E and F) Lumen-like structures formed by HUVECs at the periphery of hMSC spheroids. The scale bar represents 5 μm. (G) Lumen-like structure outside an hMSC spheroid (image only shows HUVECs). The scale bar represents 10 μm. In (D)–(G), the typical cytoplasmic material inside these lumens is indicated by the red thick arrows. In (D), WPBs within a HUVEC are indicated by the red dashed arrow. (H–K) Ultrastructural features suggesting HUVEC interaction with and infiltration into hMSC spheroids cultured on porous microwell arrays. (H) Multi-layer arrangement of HUVECs at the periphery of an hMSC spheroid after 1 day of culture. The scale bar represents 1 μm. (I) Collagen deposition, indicated by the red empty arrowheads, at the periphery of a HUVEC-vascularized hMSC spheroid after 3 days of culture. The scale bar represents 1 μm. (J) Presence of PVs, indicated by the red thin arrows, within the spheroids after 1 day of culture. The scale bar represents 500 nm. (K) Presence of secretory pod-like structures, indicated by red hashtags after 1 day of culture. The scale bar represents 1 μm.

TEM imaging of the HUVEC-hMSC spheroid co-culture samples was performed for in-depth investigation of the interaction between the two cell populations and assessing HUVECs' infiltration to form a vasculature within the spheroids. As a first step, the identification of the HUVECs, to distinguish them from the hMSCs, was performed by looking for some of the unambiguous ultrastructural features these cells present under an electron microscope. These included the observation of well-formed tight-junctions (TJs) [81] (Fig. 8A, indicated by the red circle), pinocytotic vesicles (PVs) [34] (Fig. 8B, indicated by the red thin arrows), which are responsible for transendothelial transport by eventually assembling into transport channels [82], autophagosomes (Fig. 8C, indicated by the red arrowheads), round or electron-dense Weibel-Palade bodies (WPBs) (Fig. 8C, indicated by the red dashed arrows) and secretory pods (Fig. 8C, indicated by the red hashtag), which are considered like endothelial-specific secretory granules [83].

The electron microscopy analysis of the HUVEC-vascularized hMSC spheroids provided interesting findings on the interactions of the two cell types within the microwell arrays. For the co-culture in non-porous microwells, HUVEC-lined luminal structures were visible inside the spheroid (Fig. 8D, indicated by the red asterisk), on its outer surface (Fig. 8E, indicated by the red asterisk) and at its periphery protruding outwards (Fig. 8F and G and Supplementary Fig. 8A and B, indicated by the red asterisks). Within the lumen, the presence of typical HUVEC cytoplasmic material (Fig. 8D–G, indicated by the red thick arrows) was observed, as previously reported [34,84]. Concerning the structure of the protruding sprouts, elongated HUVEC sprouts presenting a narrow luminal space have been previously described on Matrigel coats [84], which is in line with our observation of such structures in diluted Matrigel (Fig. 8G). The combination of HUVEC linings on the inner surfaces of non-porous microwells and hMSC spheroids within the same resulted in the successful generation of vascularized spheroids, which presented hollow vascular structures inside, on the surface and at the periphery of the spheroid.

For the HUVEC-vascularized hMSC spheroids cultured on porous microwell arrays, the outcomes describing the quality of the spheroid vascularization were more elusive. Penetration of capillary-like structures into the spheroid could not be clearly observed directly, although HUVEC layers were present on the surface of the hMSC spheroids (Fig. 8H), indicating direct contact between the two cell populations. The difference in infiltration compared to non-porous microwell arrays (Fig. 8D) might have been due to the delay of the vascularization process on the porous microwell arrays discussed in sections 3.3, 3.4. On the other hand, several signs of HUVEC infiltration into and interaction with the spheroids could be observed. Collagen and a basement membrane-like structure were visible near HUVECs (Fig. 8I, indicated by the red empty arrowheads), as similarly reported in literature [85]. Collagen is one of the major ECM players involved in the formation of vascular tubular structures *in vitro* [86]. Some HUVEC-typical ultrastructural features like PVs (Fig. 8J, indicated by the red thin arrows) and secretory pods (Fig. 8K, indicated by the red hashtags) were also observed inside the spheroids. Although less conclusive than for the non-porous microwells, all these observations suggest a promising cooperation of HUVECs and hMSC spheroids as building blocks of vascularized 3D cell aggregates. Moreover, microwell-based vascularization using porous microwells was overall slower compared to non-porous ones throughout all the experiments of this study, which may also explain the less evident vascularization inside the spheroids at the time of the current analysis.

## 4 Conclusions and outlook

We presented a new approach for vascularizing 3D cell aggregates *in vitro*. The proposed approach was based on the culture of endothelial cells, in this case HUVECs, on non-porous and porous microwell arrays as vascular beds from which sprouting can occur. The microwells were fabricated by microthermoforming from dense and porous films. The optimal pore diameter of the porous films was found to be 0.8 μm, which resulted in pores in the microwell walls of on average up to around 4.24 μm in diameter. The HUVECs adhered to and progressively colonized both non-porous and porous microwells over the course of 4 days in culture. When adding a dilution of Matrigel to the microwells, the HUVECs re-arranged into sprout-like 3D structures within the microwell space. The quantification of a selection of vascular markers, such CD31, laminin and podocalyxin, as well as the characterization of the sprout number and length indicated a cell behavior toward the formation of a functional vasculature particularly after 1 day in culture. As a further confirmation of this, a FITC-dextran diffusion assay was performed to identify the sprouts as luminal.

To showcase the versatility of the microwell-integrated approach, we branched out to set up a lymphangiogenesis model. For this, we adapted the microwell-based culture to the biochemical requirements of the sprouting of hDLECs, which successfully occurred after 1 day in culture under a VEGF-C gradient in the non-porous microwells.

As a proof of concept, we cultured the HUVECs together with hMSC or MG-63 spheroids on the microwell arrays, to evaluate the formation of capillary-like structures around and in the 3D cell aggregate. Abundant outer vascularization of the hMSC spheroids was verified by visualizing and quantifying the expression of relevant vascular markers while for the presence of an inner vascularization of the spheroids this was done via TEM. In the latter case, the co-culture in non-porous microwells outperformed the co-culture in porous ones. For the tumor spheroids, more evident signs of inner vascularization could be observed compared to the stromal spheroids.

Outlooks for the proposed vascularization approach foresee the study and enabling of longer durations of the (co-)culture of the spheroids in the HUVEC-covered microwells to investigate the self-organization, remodeling and maintenance of the hybrid system at later stages. This should then also include the further characterization of the engineered sprouting network by assessing its protein composition and gene expression over the course of its maturation. From a fundamental point of view, a clearer and more systematic understanding of the vessels functionality when actively perfused at physiological flow levels would also be necessary. This would be important for the characterization of the vascular network per se, but also for assessing its active role of functionally nourishing target spheroids. In this context, our microwell arrays could already be shown to be compatible with perfused 3D cell culture in microfluidic bioreactors [87]. Future uses of the proposed *in vitro* vascularization approach can explore the manifold possibilities that the film or membrane-based microwell arrays can thereby offer. This could include other, for example, stadium- or channel-type microwell shapes and variations of the diameters, densities and possibly also locations of the pores in the microwell walls. Furthermore, the microwell arrays could be integrated into microfluidic culture platforms to add and maintain relevant physiological stimuli such as shear stress and gradients of soluble factors, respectively.

In terms of future applications, the platform might lend itself to the efficient vascularization of other mini- and microtissues, such as skeletal muscle spheroids, or lung, kidney or intestinal (cystic) organoids. The platform would then allow to study the process of vascularization or the vascularized tissues themselves, also in comparison with non-vascularized ones. The platform could be used for fundamental physiology studies, as well as for testing/screening of molecules or even (micro)biomaterials [29]. Future research may also lean toward the use of the microwell arrays as a platform to study and engineer innervation *in vitro*, also as there is a tight interconnection and cross-talk between blood vessels and nerves [88], which is yet to be fully elucidated. Last but not least, the platform might also be useful for forming and engineering large numbers of pre-vascularized spheroids to then harvest and use them for implantable tissue regeneration solutions, for example. After implantation, the engineered pre-mature networks could then anastomose with the host vasculature.

## CRediT authorship contribution statement

**Maria G. Fois:** Writing – review & editing, Writing – original draft, Visualization, Validation, Methodology, Investigation, Formal analysis. **Zeinab N. Tahmasebi Birgani:** Writing – review & editing, Visualization, Validation, Supervision, Methodology, Conceptualization. **Carmen López-Iglesias:** Writing – review & editing, Visualization, Validation, Methodology, Investigation, Formal analysis. **Kèvin Knoops:** Writing – review & editing, Visualization, Methodology, Investigation. **Clemens van Blitterswijk:** Writing – review & editing, Funding acquisition. **Stefan Giselbrecht:** Writing – review & editing, Supervision, Methodology, Funding acquisition, Conceptualization. **Pamela Habibović:** Writing – review & editing, Supervision, Methodology, Funding acquisition, Conceptualization. **Roman K. Truckenmüller:** Writing – review & editing, Validation, Supervision, Methodology, Funding acquisition, Conceptualization.

## Declaration of competing interest

The authors declare the following financial interests/personal relationships, which may be considered as potential competing interests: Roman K. Truckenmueller reports a relationship with 300MICRONS GmbH that includes: board membership and equity or stocks. Stefan Giselbrecht reports a relationship with 300MICRONS GmbH that includes: board membership and equity or stocks. Roman K. Truckenmueller has the patent #Moulded bodies, method for producing said bodies and use thereof, EP20050757982, licensed to 300MICRONS GmbH. Stefan Giselbrecht has the patent #Moulded bodies, method for producing said bodies and use thereof, EP20050757982, licensed to 300MICRONS GmbH. Roman K. Truckenmueller and Stefan Giselbrecht are (co-)founders, shareholders and managing directors of 300MICRONS GmbH. Roman K. Truckenmueller and Stefan Giselbrecht are (co-)inventors of the patent ‘Moulded bodies, method for producing said bodies and use thereof, EP20050757982’; holder of the patent is Karlsruhe Institute of Technology. Roman K. Truckenmueller and Stefan Giselbrecht are members of the Strategic Advisory Board of ReGEN Biomedical B.V. If there are other authors, they declare that they have no known competing financial interests or personal relationships that could have appeared to influence the work reported in this paper.

## Data Availability

Data will be made available on request.
