# Supplementary material for: In vitro vascularization of 3D cell aggregates in microwells with integrated vascular beds
Source: Mater Today Bio. 2024 Sep 19;29:101260. doi: 10.1016/j.mtbio.2024.101260 (PMC11466645; doi:10.1016/j.mtbio.2024.101260)
Supplement: Multimedia component 1 [file mmc1.docx]

**Supplementary data**

***In vitro* vascularization of 3D cell aggregates in microwells with integrated vascular beds**

Maria G. Fois, Zeinab N. Tahmasebi Birgani, Carmen López-Iglesias, Kèvin Knoops, Clemens van Blitterswijk, Stefan Giselbrecht†, Pamela Habibović†, Roman K. Truckenmüller†*

†these authors equally contributed to this work

*Correspondence to: [r.truckenmuller@maastrichtuniversity.nl](mailto:r.truckenmuller@maastrichtuniversity.nl)


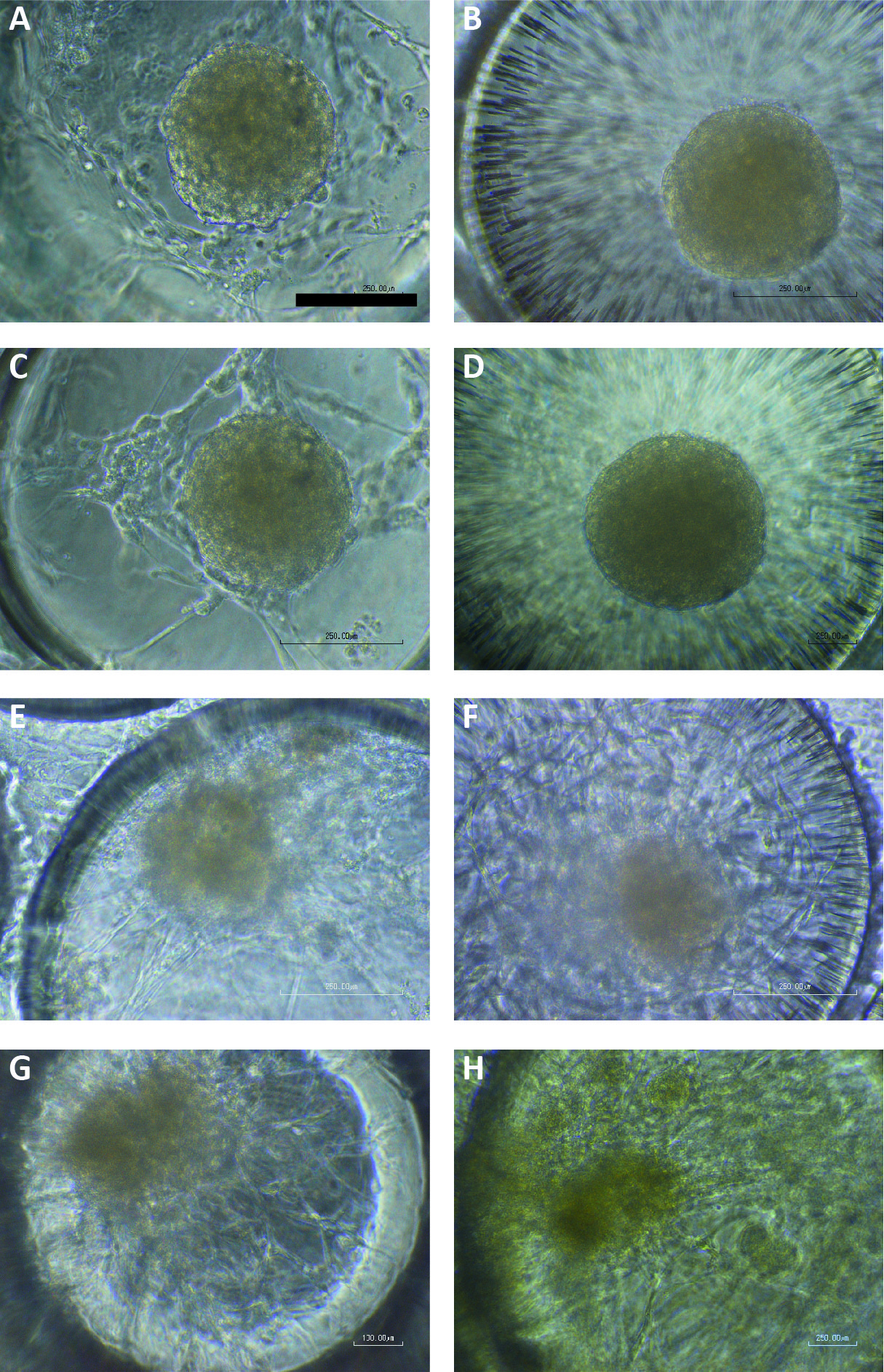


**Supplementary fig. 1. hMSC spheroids in HUVEC-covered microwells for culture durations of up to 7 days:** (A–H) The images show (co )cultures on non-porous and porous microwells (left and right column, respectively) and for 30 min, 6 h, 5 days and 7 days (1^st^, 2^nd^ , 3^rd^ and 4^th^ row, respectively).


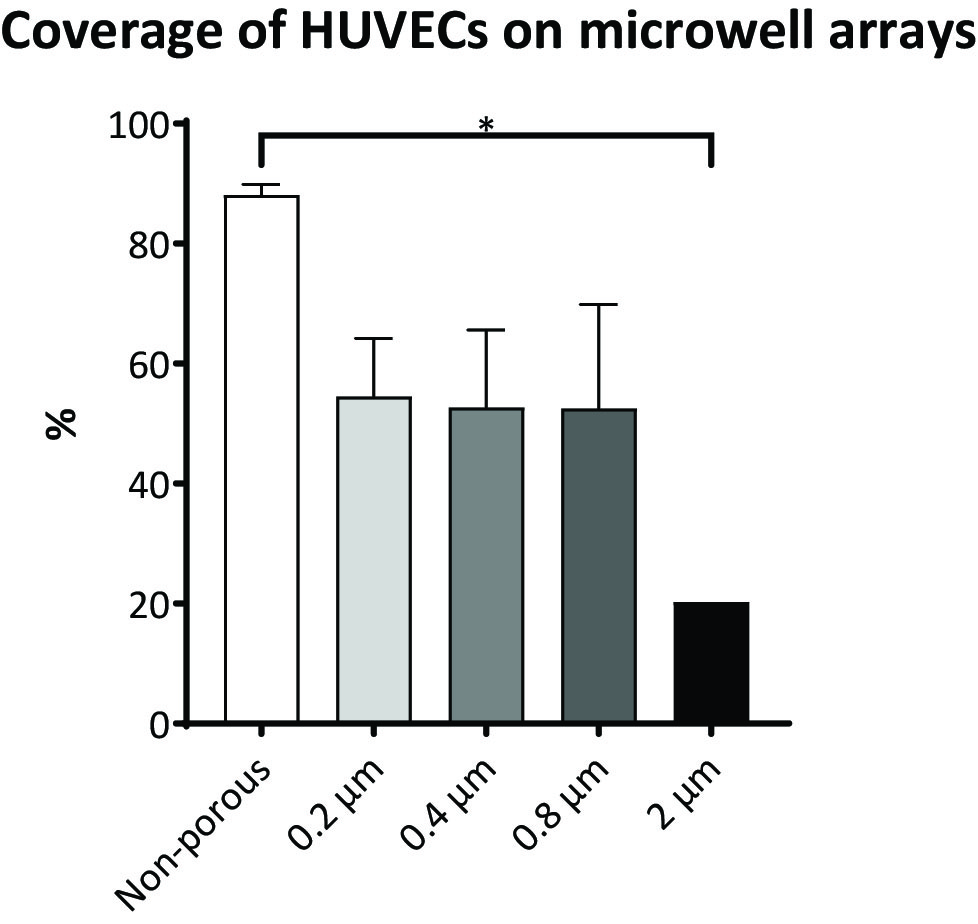


**Supplementary fig. 2. Quantification of the percentage of the microwell area covered by adhering HUVECs in dependence of the original films used for their fabrication as area coverage by image analysis in CellProfiler:** The analysis was performed on maximum projection images not considering the curved shape of the microwells performed after 4 days in culture in EGM2. The statistical method used here was a one-way ANOVA and “*” indicates a p < 0.05.


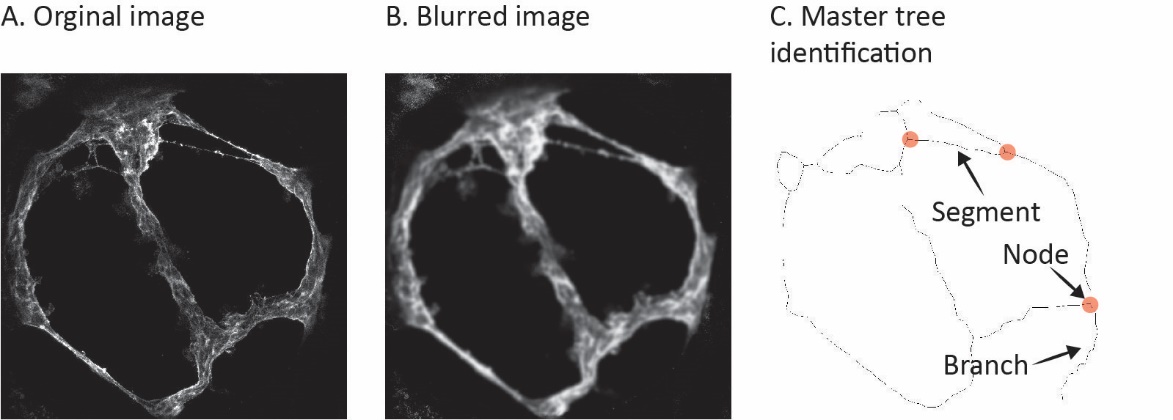


**Supplementary fig. 3. Sprout number and length quantification:** Example of the adapted *Angiogenesis Analyzer* plugin to quantify sprout number and length using the CD31 images. (A) Original image. (B) Blurred image. (C) Master tree identification. In this step, the skeletonized structure of the vascular tree was analyzed after recognizing its key elements, such as nodes, segments and branches. The total number and length of the sum of branches and segments was considered as parameters to evaluate angiogenesis on the microwell arrays.


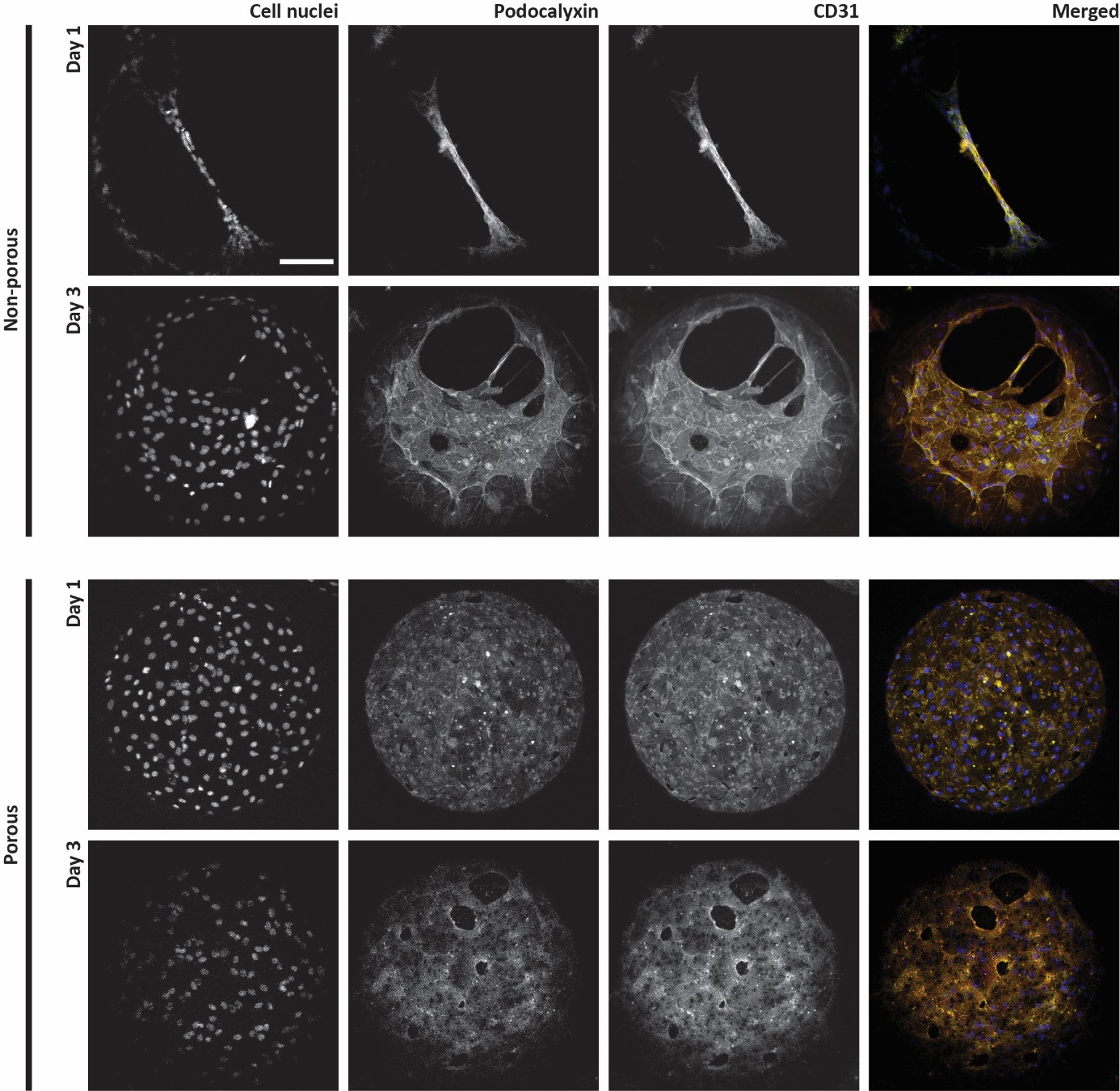


**Supplementary fig. 4. hDLEC culture and sprouting on the microwell arrays:** Maximum intensity projections of confocal fluorescence microscopy images of hDLECs on non-porous and porous microwell arrays after 1 and 3 days of culture in 50% Matrigel in EMV2 . Cell nuclei, podocalyxin and CD31 are stained in blue, green and red, respectively (shown only in the merged images). The scale bar represents 100 µm and applies to all images.


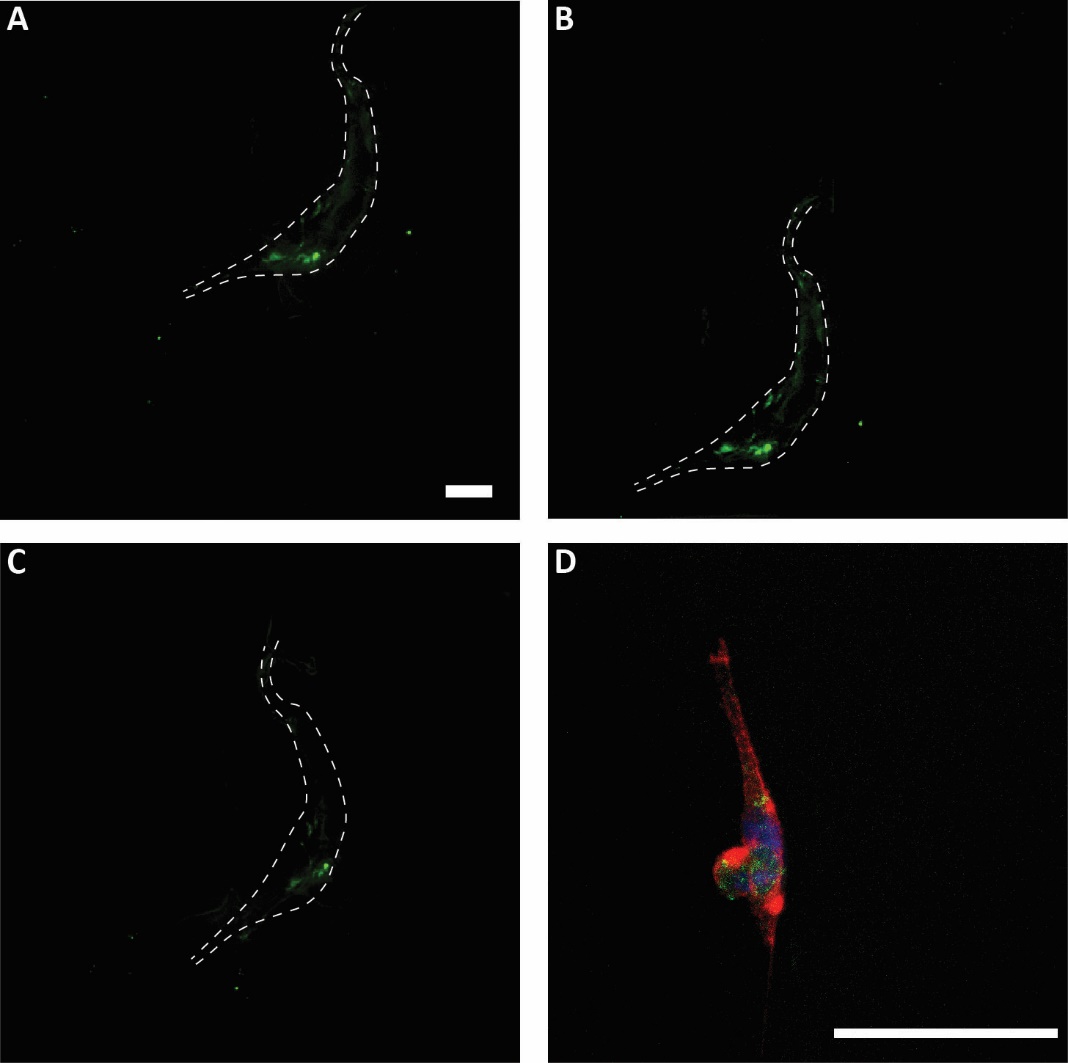


**Supplementary fig. 5.** **Vascular permeability assessment through FITC-dextran diffusion.** (A–C) Fluorescence microscopy images of HUVECs on porous microwell arrays after first 20 min of culture in 50% Matrigel in EGM2 and then (A) 5, (B) 10 and (C) 20 min of FITC-dextran diffusion. The scale bar represents 100 µm and applies to the subfigures (A)–(C). (D) Maximum intensity projections of confocal fluorescence microscopy images of HUVECs on porous microwell arrays after 3 days of culture in 50% Matrigel in EGM2. FITC-dextran within the capillary-like structure appears in green and cell nuclei and cytoskeletal actin are stained in blue and red, respectively. The scale bar represents 50 µm.


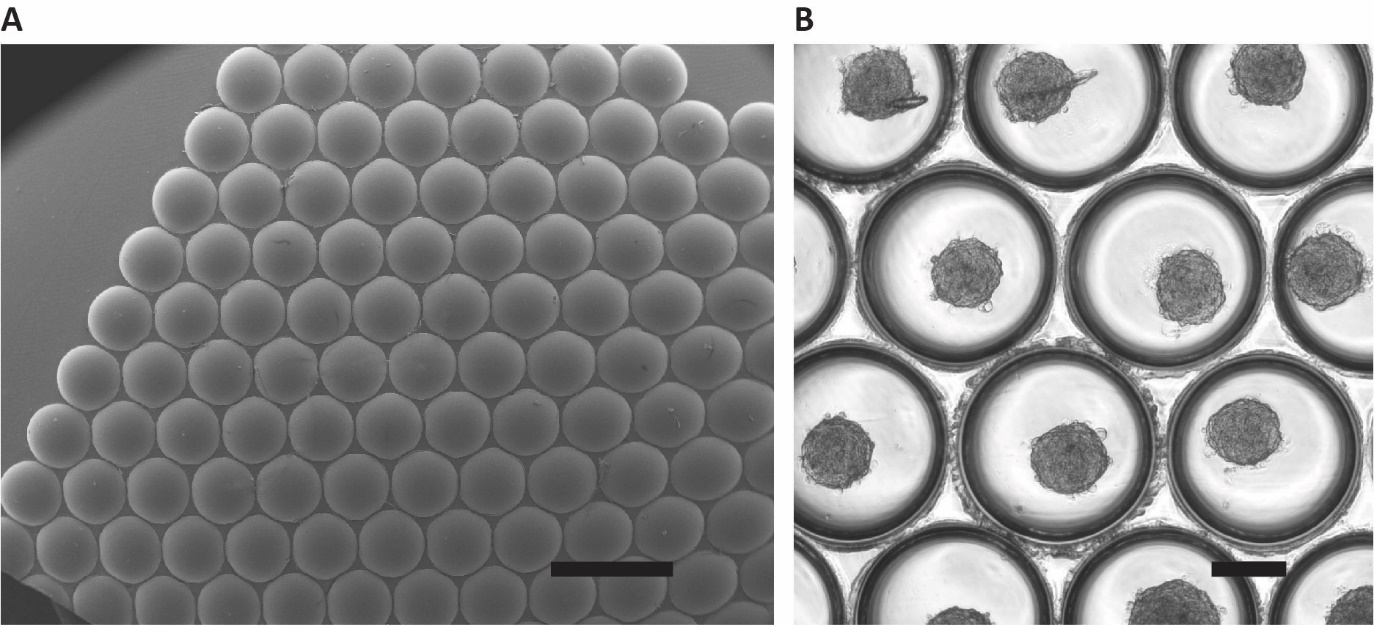


**Supplementary fig. 6. hMSC spheroid culture and formation:** (A) Back/bottom view SEM image of (section of) non-porous 289-microwell array. The scale bar represents 1 mm. (B) Bright-field microscopy image of hMSC spheroids in 289-microwell array after 48 h in culture. The scale bar represents 200 µm.


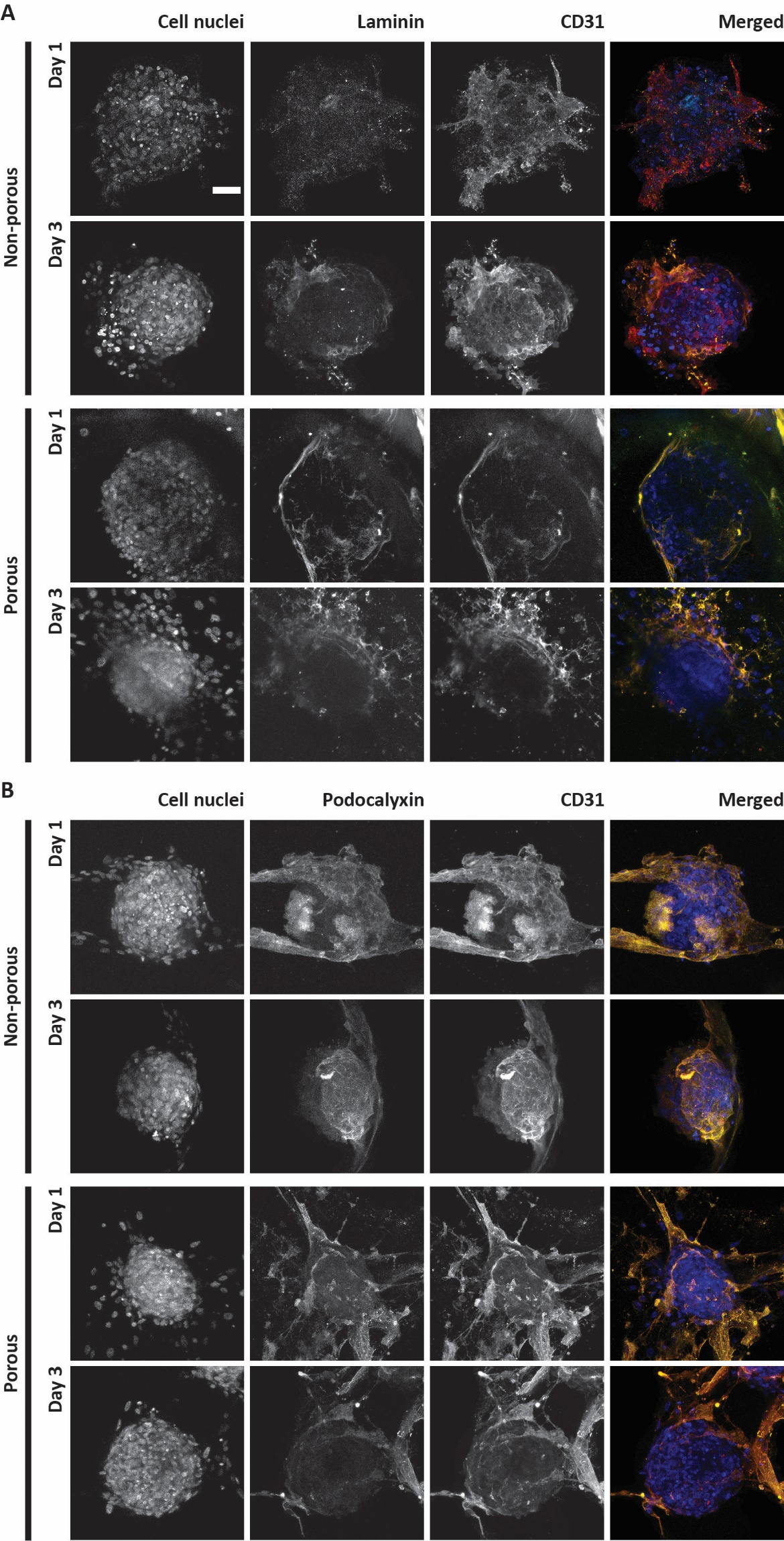


**Supplementary fig. 7. MG-63 spheroid vascularization:** Maximum intensity projections of confocal fluorescence microscopy images of co-cultures of MG-63 spheroids and HUVECs on microwell arrays after 1 and 3 days of culture in 50% Matrigel in co-culture medium. (A) Basement membrane visualization of HUVEC-vascularized MG-63 spheroids cultured on non-porous and in porous microwell arrays. Cell nuclei, laminin and CD31 are stained in blue, green and red, respectively (shown only in the merged image). The scale bar represents 50 µm and applies to all images. (B) Luminal podocalyxin visualization of HUVEC-vascularized MG-63 spheroids cultured on non-porous and in porous microwell arrays. Cell nuclei, podocalyxin and CD31 are stained in blue, green and red, respectively (shown only in the merged images).


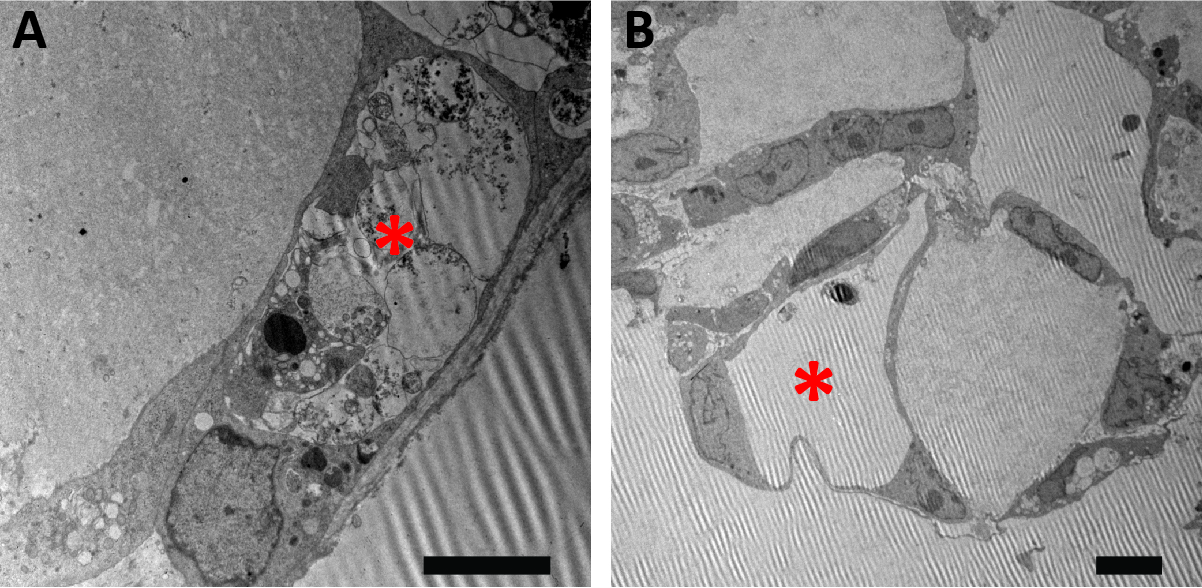


**Supplementary fig. 8: EM of HUVEC-vascularized hMSC spheroids after 3 days of culture on non-porous microwell arrays in 50% Matrigel in co-culture medium:** (A and B) Evidences of formation of HUVEC-lined lumen-like structures outside hMSC spheroids (images only show HUVECs), indicated by the red asterisks. The scale bars represent 5 and 10 µm, respectively.


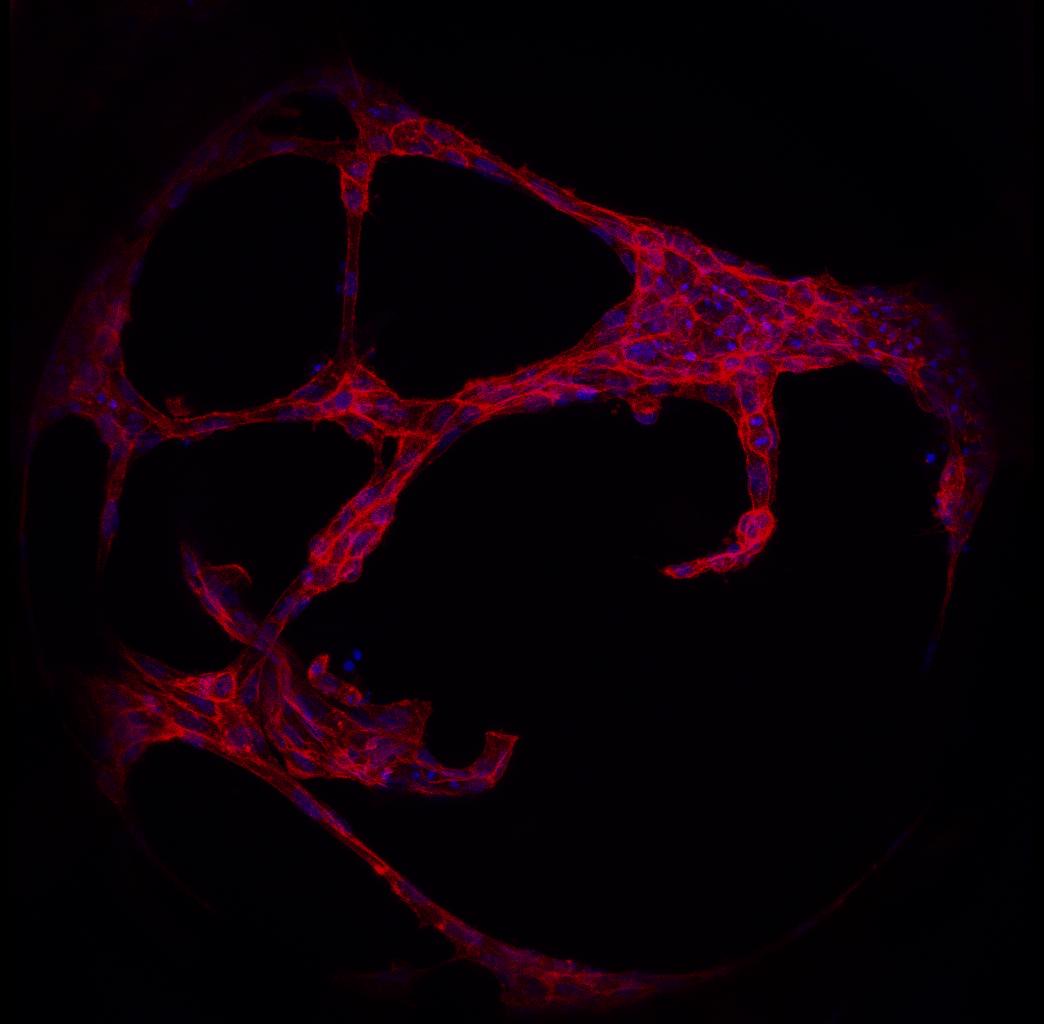

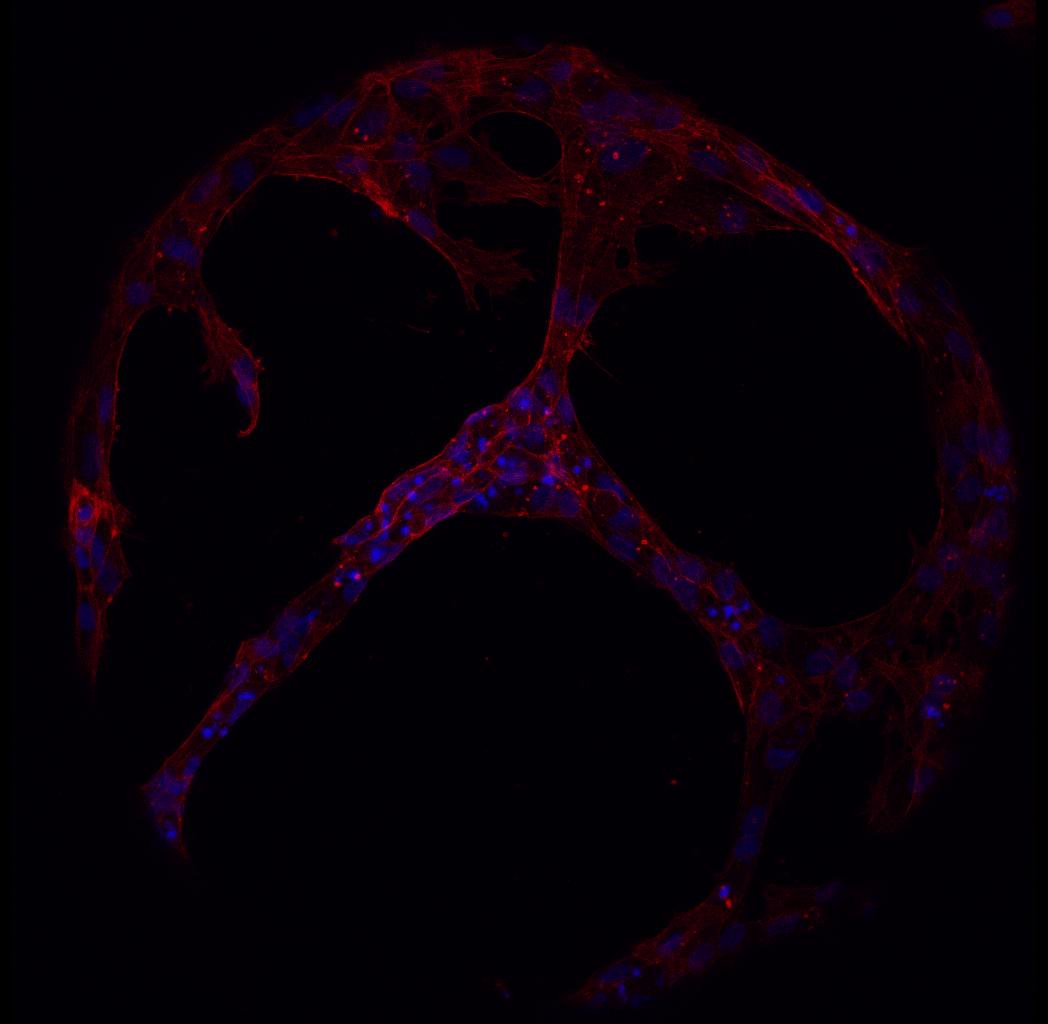

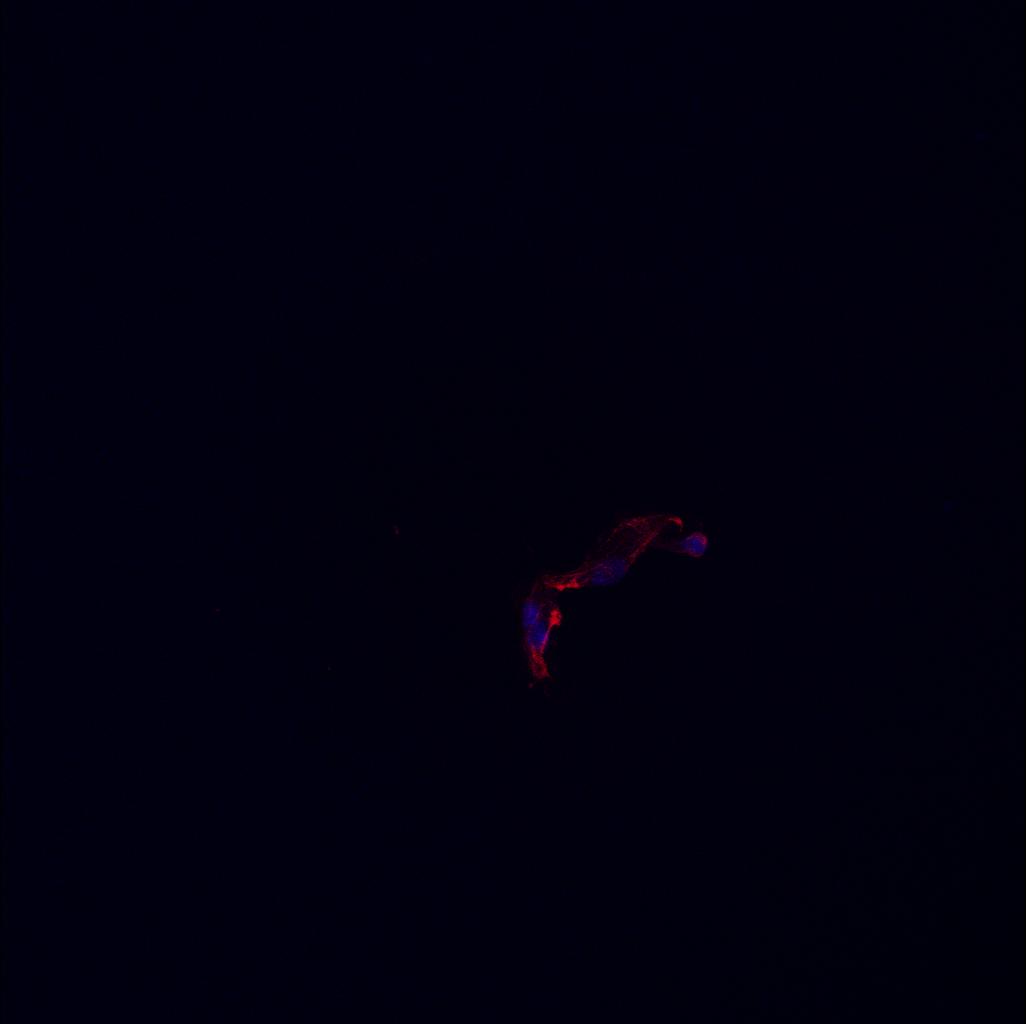

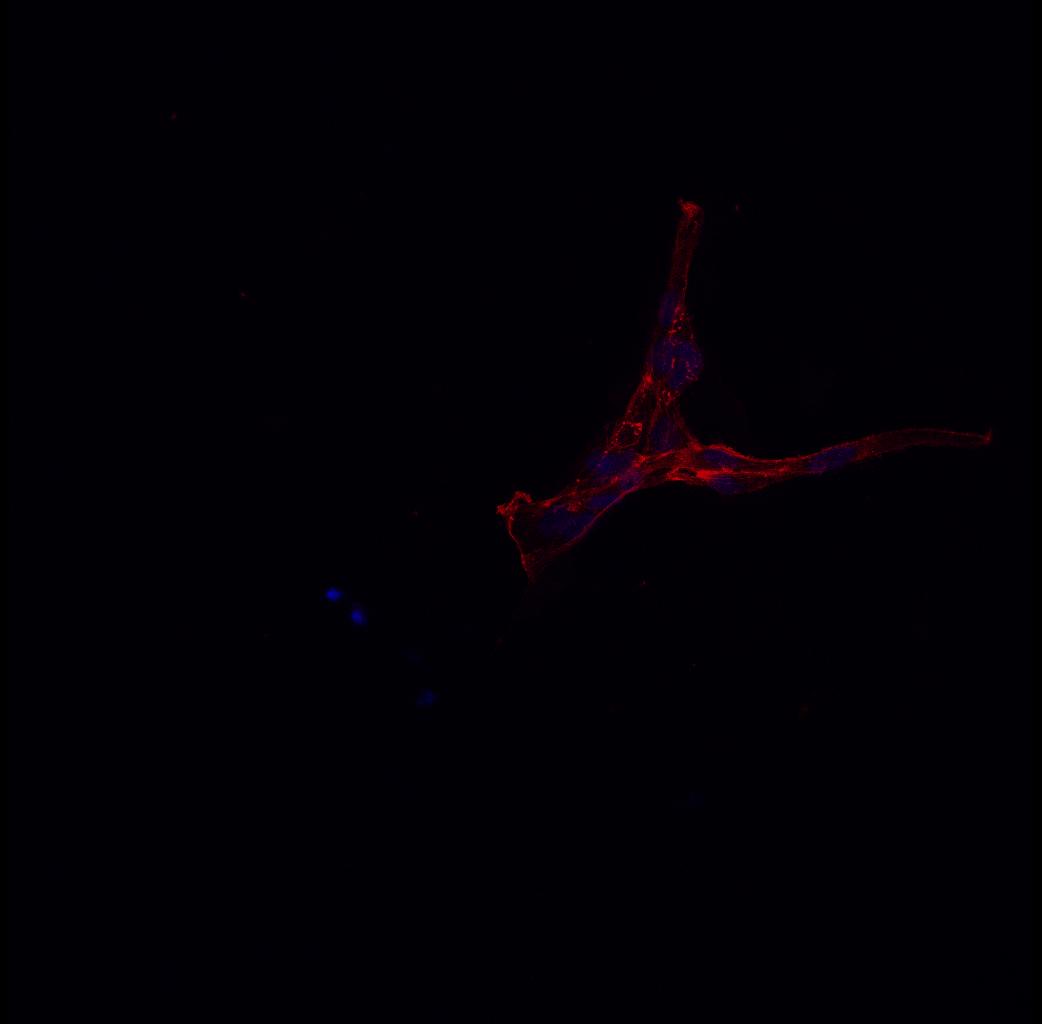

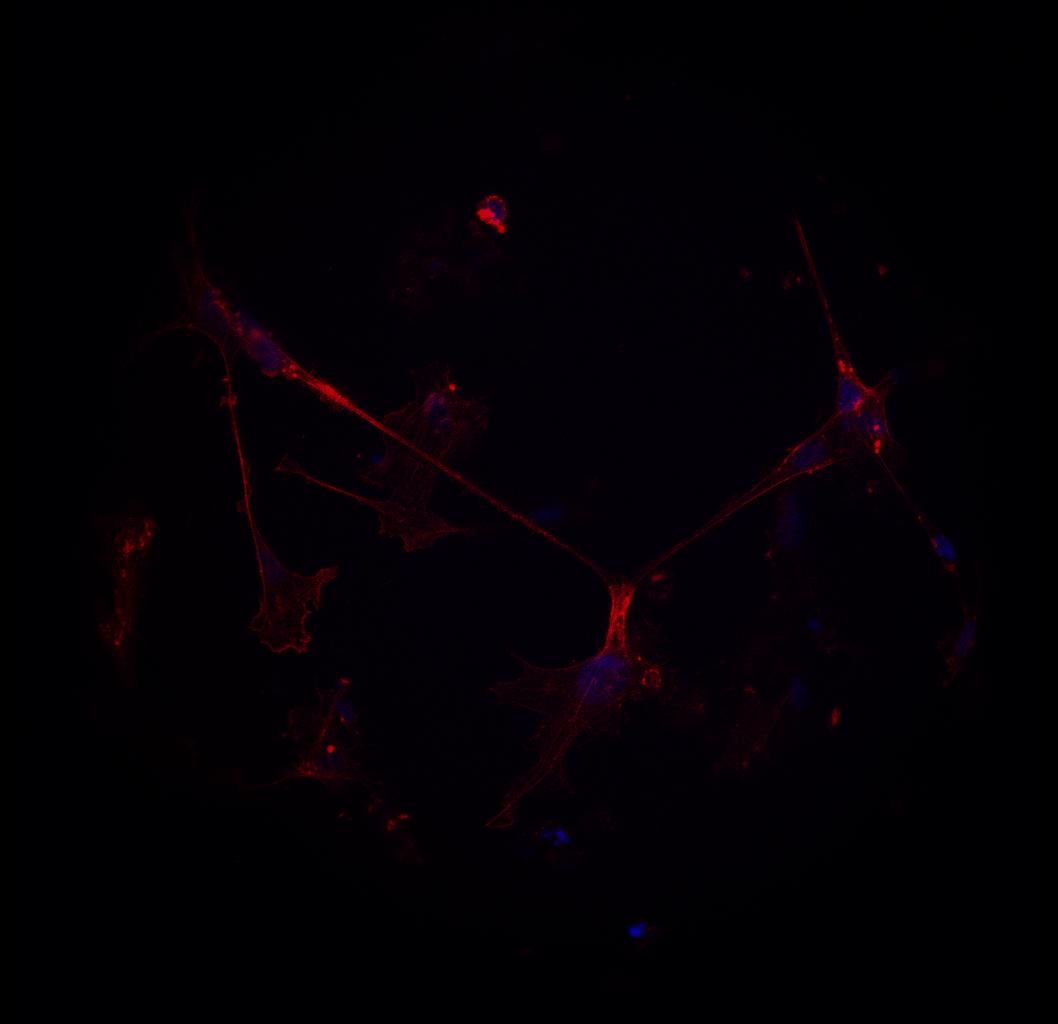


E

D

C

B

A

**Supplementary video 1. 3D reconstruction of a representative sprouted HUVEC network in microwells:** (A) HUVEC 3D sprouting network in non-porous microwells after 1 day of culture in 50% Matrigel in EGM2. (B–E) HUVEC 3D sprouting network in microwells made from the (B) 0.8, (C) 0.2, (D) 0.4 and (E) 2 µm-pore films after 1 day of culture in 50% Matrigel in EGM2. In (A)–(E), cell nuclei and actin are stained in blue and red, respectively; the width and height of the frames are both 465.05 µm.


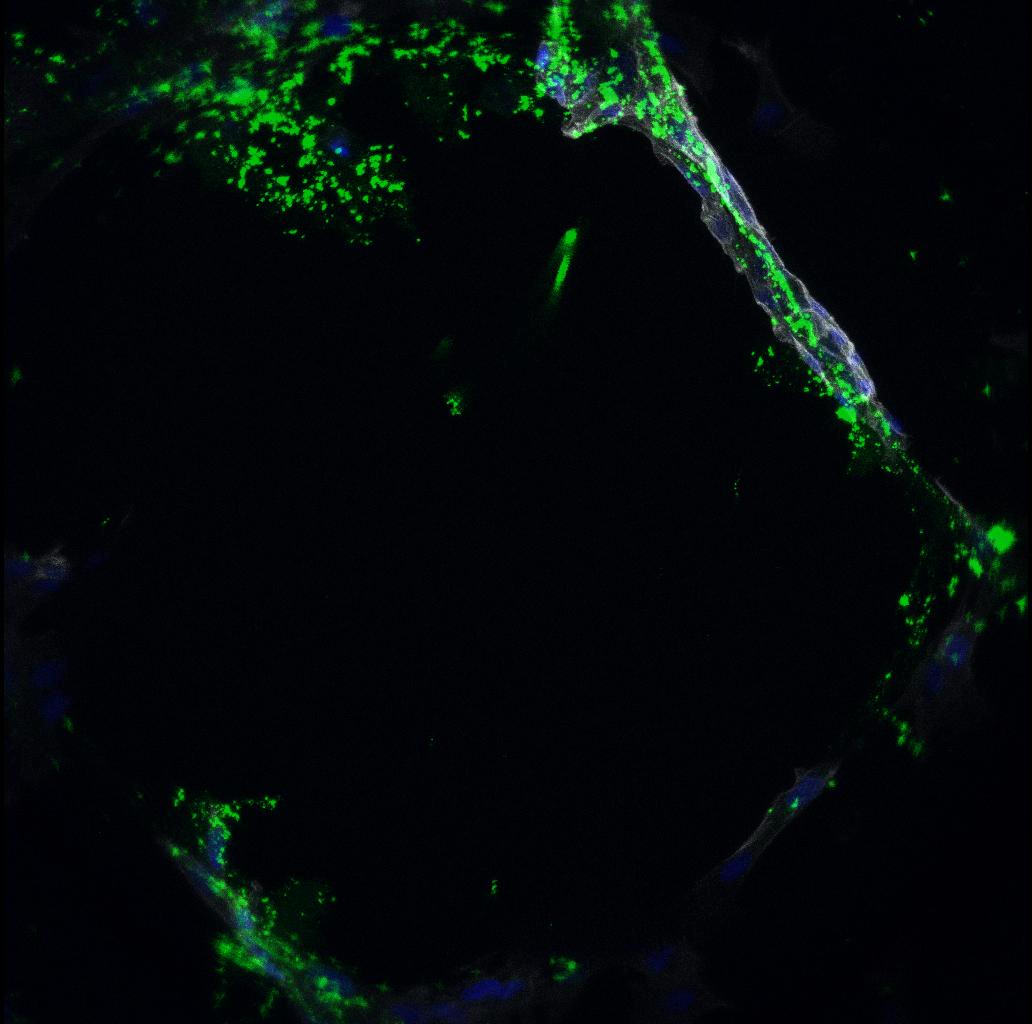


**Supplementary video 2.** **3D reconstruction of a representative sprouted HUVEC network in non-porous microwells:** HUVEC 3D sprouting network after 3 days of culture in 50% Matrigel in EGM2. Cell nuclei, actin and luminal podocalyxin are stained in blue, grey and green, respectively. The width and height of the frame are both 465.05 µm.


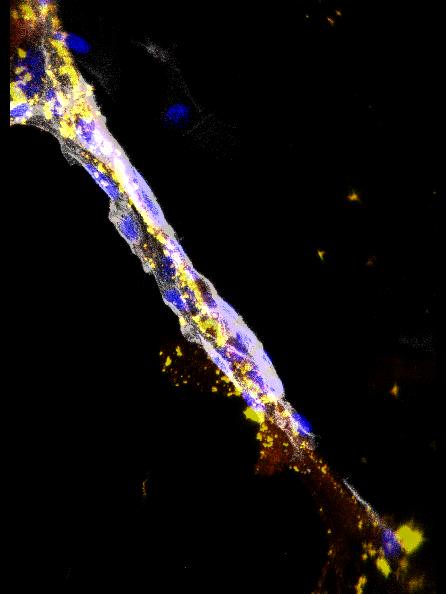


**Supplementary video 3. (Section of a) 3D reconstruction of a representative sprouted HUVEC network in non-porous microwells:** Higher magnified version of the 3D reconstruction of Supplementary video 2 with additional staining of CD31. Cell nuclei, actin, CD31 and luminal podocalyxin are stained in blue, grey, red and green, respectively. The width and height of the frame are 192.69 and 268.69 µm, respectively.


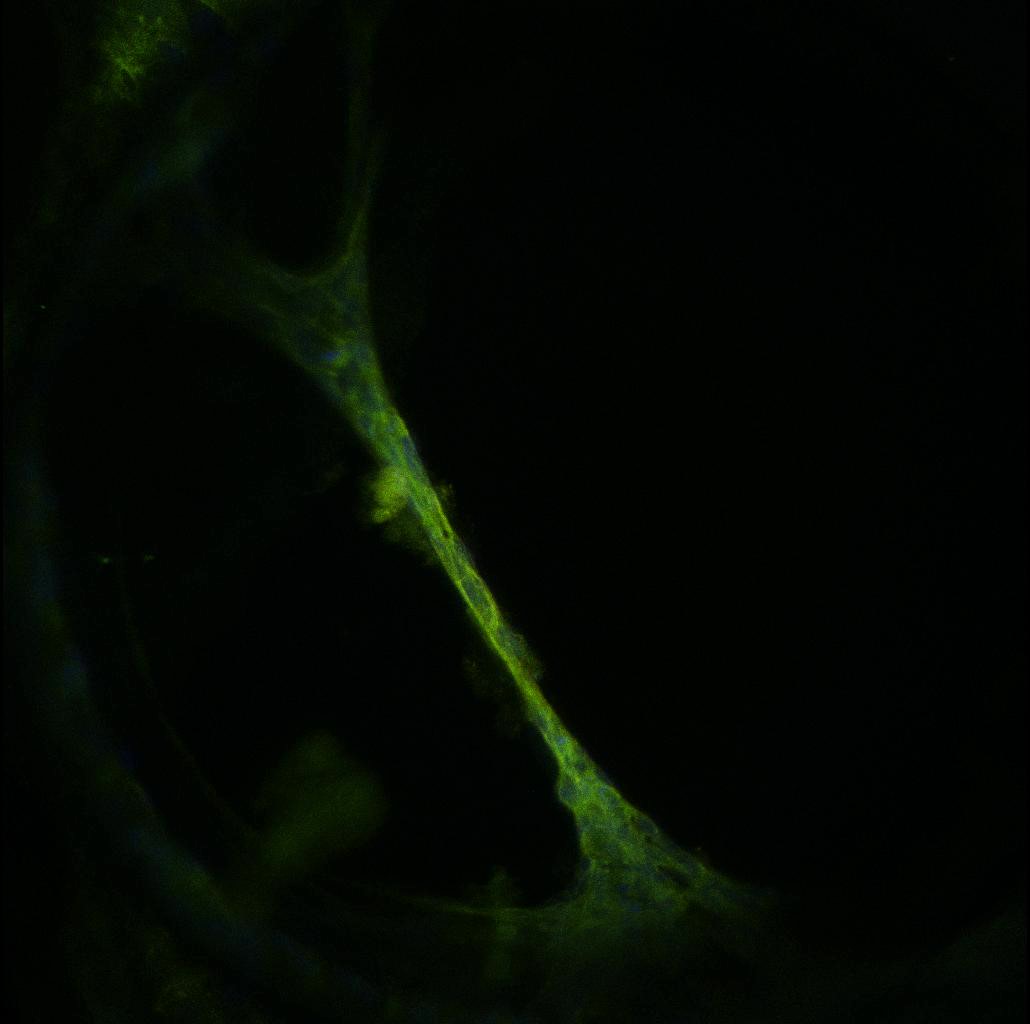

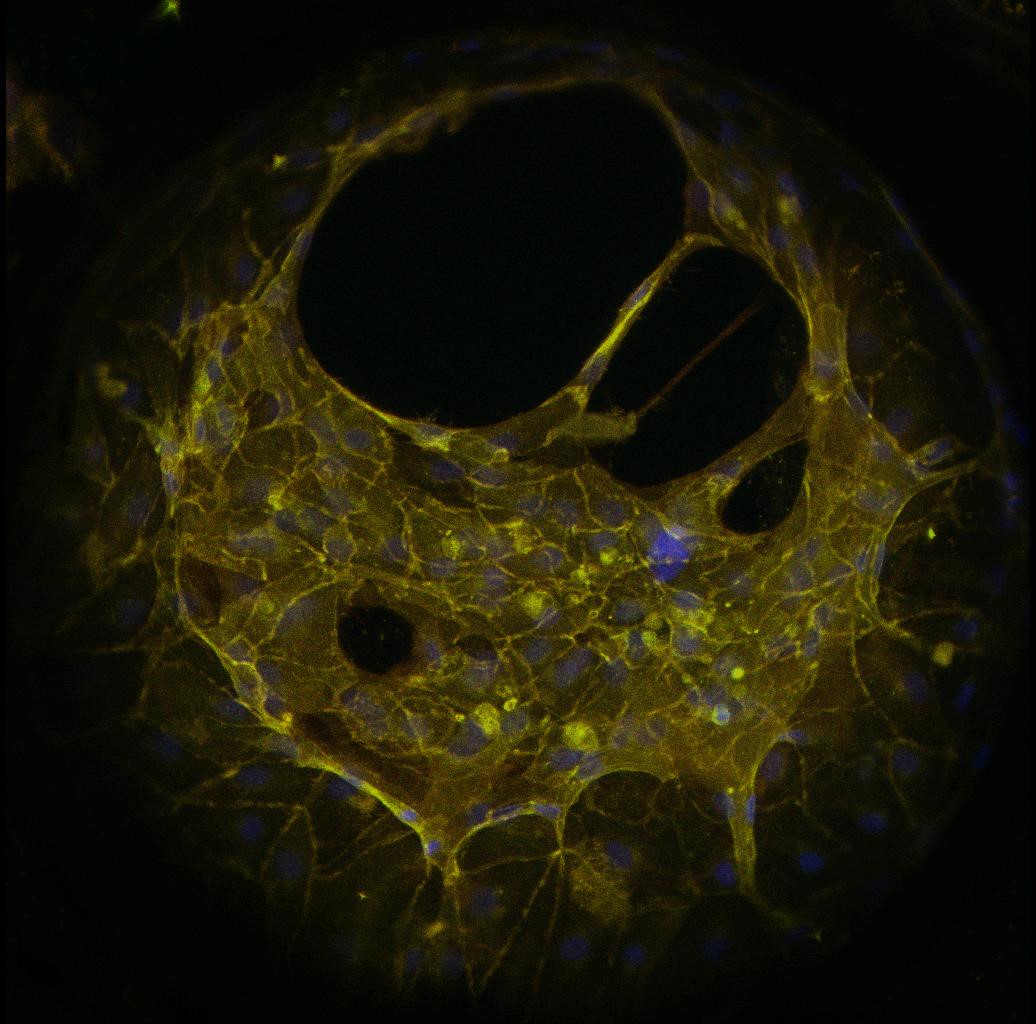


B

A

**Supplementary video 4.** **3D reconstruction of a representative sprouted hDLEC network in non-porous microwells:** hDLEC 3D sprouting network after (A) 1 and (B) 3 days of culture in 50% Matrigel in EMV2. Cell nuclei, luminal podocalyxin and CD31 are stained in blue, green and red, respectively. The width and height of the frames are both 465.05 µm.
